# A literature-based review of Hymenoptera Parasitica and Chrysidoidea from Reunion Island

**DOI:** 10.3897/zookeys.652.10729

**Published:** 2017-02-06

**Authors:** David Muru, Michael Madl, Maxime Jacquot, Jean-Philippe Deguine

**Affiliations:** 1 CIRAD, UMR PVBMT, F – 97410 Saint-Pierre, La Réunion, France; 2 Naturhistorisches Museum, 2 Zoologische Abteilung, Burgring 7, 1010 Wien, Austria; 3 Université de La Réunion, UMR PVBMT, F-97410, Saint-Pierre, La Réunion, France

**Keywords:** Hymenoptera, checklist, Chalcidoidea, Ichneumonoidea, Diaprioidea, Cynipoidea, Evanioidea, Chrysidoidea, Ceraphronoidea, Platygastroidea, Reunion Island

## Abstract

A review of the genera and species of Hymenoptera Parasitica and Chrysidoidea reported so far from Reunion Island is provided with host information. Data presented here is based on a review of the existing literature by the authors. The list includes: (1) 156 species of Ichneumonoidea belonging to 65 genera and 25 subfamilies (Braconidae: Agathidinae, Alysiinae, Aphidiinae, Braconinae, Charmontinae, Cheloninae, Doryctinae, Euphorinae, Gnamptodontinae, Microgastrinae, Opiinae; Ichneumonidae: Banchinae, Campopleginae, Cremastinae, Cryptinae, Diplazontinae, Ichneumoninae, Mesochorinae, Metopiinae, Ophioninae, Orthocentrinae, Pimplinae, Tersilochinae, Tryphoninae); (2) 121 species of Chalcidoidea belonging to 56 genera and 8 families (Agaonidae, Aphelinidae, Chalcididae, Encyrtidae, Eulophidae, Eupelmidae, Eurytomidae, Ormyridae, Pteromalidae, Signophoridae, Torymidae and Trichogrammatidae); (3) seven species of Cynipoidea (family Figitidae); (4) six species of Chrysidoidea in three families (Bethylidae, Chrysididae, Dryinidae); (5) five species of Platygastroidea (families Platygastridae and Scelionidae); (6) five species of Diaprioidea (family Diapriidae); (7) four species of Ceraphronoidae (families Ceraphronidae and Megaspilidae); and (8) two species of Evanioidea (family Evaniidae). This review records a total of 306 species.

## Introduction

The parasitoids of the Hymenoptera Apocrita form one of the most species-rich groups of animals, potentially representing more than 20% of the world’s insects (LaSalle and Gauld 1992). Furthermore, they play an important role in the regulation of insect pests and many of them are used in biological control programs all over the world. The failures and successes of their use in biocontrol have been extensively reviewed (e.g. DeBach 1964, Funasaki et al. 1988, DeBach and Rosen 1991, van Driesche and Bellows 1996, Smith 1996, Solomon et al. 2001).

To date, the diversity of Hymenoptera in La Réunion has been partially studied. There have been some studies on the hymenopteran diversity around 1950. Risbec (1957) and Benoit (1957) reviewed the Chalcidoidea/Proctotrupoidea and the Ichneumonoidea, respectively. To our knowledge, after 1957 almost nothing was published on the hymenopteran diversity of Reunion Island (only Wiebes 1981 on the figwasps) untill 2001 when Vayssière et al. published a review of the pests and their natural enemies on several crops of the island. Two years later, this work was followed and updated by the publication of a book (Quilici et al. 2003). At this time, taxonomic work on Parasitica was far from exhaustive because it only dealt with parasitoids associated with important pests. In fact, a lot of work has been done to combat key pests such as Tephritid flies (Diptera: Tephritidae) in mango and cucurbit fruits. Nevertheless, the taxonomic review of the fauna from Reunion Island has been conducted for a few groups. Rousse and Villemant (2012) reviewed the Ichneumonidae and provided a key to species of the Island with 15 species new to science. Some subfamilies of Braconidae (Ichneumonoidea) have been reviewed: Madl (2007, Agathidinae), Fischer and Madl (2008, Opiinae), Rousse and Braet (2012, Euphorinae), Braet et al. (2012, Cheloninae), Rousse and Gupta (2013, Microgastrinae). Madl and van Achterberg (2014) published a catalogue of the Braconidae of Malagasy subregion, including Reunion Island. Fischer (2014) published an important paper on Alysiinae and Opiinae (Braconidae) with 12 species new to science. Until to now the study of Ichneumonoidea has added more than 140 species to the list of Benoit (1957).

Superfamilies other than the Ichneumonoidea were less studied. For example, the Chalcidoidea may have a diversity similar to or higher than that of the Ichneumonoidea. In fact, there are a lot of unpublished data from the work of Marc Attié (2001–2004). The material collected during his work is now at the CBGP in Montpellier (France).

By reviewing the published data, this work provides a first list of all the Hymenoptera Parasitica of Reunion Island and the superfamily Chrysidoidea (belonging to the Hymenoptera Aculeata). We think this work will be a valuable tool for future inventories or work on biological control. Indeed, this work allows the detection of new indigenous natural enemies (e.g. *Pristomerus river*, Ichneumonidae, parasitoid of *Prophantis smaragdina*, Lepidoptera) and invasive hyperparasitoid or parasitoid of predators (e.g. *Homalotylus eytelweinii*, Encyrtidae, a parasitoid of *Rodolia chermesina*, Coccinellidae).

## Arrangement of the checklist

In this review, superfamilies are treated according to their importance, the most diverse (Ichneumonoidea) presented first. Genera known so far from Reunion Island are grouped according to subfamilies, and family affiliation. For convenience, families, subfamilies, genera and species are listed in alphabetical order. Genera and species names are followed by the names of the author(s) and year of first description, then by the reference of the record and, when available, host data.

The review includes three tables summarizing (1) the Ichneumonoidea, (2) the Chalcidoidea, (3) the records for the other superfamilies, each with general host information.

## Discussion

The present work is a literature-based review. Therefore, users should be aware that it might contain some mistakes present in the original literature. However, all references are listed so that the records can be validated.

This review lists 306 species of parasitoid Hymenoptera recorded from the island belonging to eight superfamilies. Of the species and genera reported from the island, at least 1 genus and 14 species of Ichneumonidae (Rousse and Villemant 2012), and 1 genus and 25 species of Braconidae (Madl 2007, Rousse and Braet 2012, Rousse and Gupta 2013), may be endemic as they currently only are recorded from Reunion Island. On the other hand, several species are now cosmopolitan in distribution due to their widespread use as biocontrol agents.

When compared with other geographical regions of the world, the study of hymenopteran parasitoid diversity in Reunion Island is still preliminary. Many new taxa will probably be found from the region in the future, and thus this checklist will need to be periodically updated.

## Checklist of Hymenoptera Parasitica and Chrysidoidea from Reunion Island

### Superfamily: ICHNEUMONOIDEA

#### Family: BRACONIDAE

##### Subfamily: Agathidinae

**Genus: *Biroia*** Szépligeti, 1900

*Biroia costata* (Brullé, 1846)

Recorded from: Granger 1949

Host information: Unknown

**Genus: *Camptothlipsis*** Enderlein, 1920

*Camptothlipsis curticornis* Granger, 1949

Recorded from: Quilici et al. 2003

Host information: *Nephopterix beharella* (Lepidoptera, Pyralidae) (Quilici et al. 2003, Madl 2007)

**Genus: *Coccygidium*** de Saussure, 1890

*Coccygidium lutea* (Brullé, 1846)

Recorded from: Szépligeti 1904

Host information: *Condica conducta*, *Mythimna curvula*, *Mythimna loreyi* (Lepidoptera, Noctuidae) (Madl 2007)

##### Subfamily: Alysiinae

**Genus: *Adelphenaldis*** Fischer, 2003

*Adelphenaldis grimmorum* Fischer, 2014

Recorded from: Fischer 2014

Host information: Unknown

*Adelphenaldis nanocorpus* Fischer, 2014

Recorded from: Fischer 2014

Host information: Unknown

**Genus: *Aphaereta*** Förster, 1863

*Aphaereta* sp.

Recorded from: Quilici et al. 2003

Host information: Diptera Cyclorrhapha (Gauld and Bolton 1988)

**Genus: *Asobara*** Förster, 1863

*Asobara diadegmae* Fischer, 2014

Recorded from: Fischer 2014

Host information: Hyperparasitoid of *Diadegma* sp. (ex *Plutella xylostella*, Lepidoptera) (Fischer 2014)

**Genus: *Cratospila*** Förster, 1863

*Cratospila sinenotaulis* Fischer, 2014

Recorded from: Fischer 2014

Host information: Unknown

**Genus: *Dacnusa*** Haliday, 1833

*Dacnusa sibirica* Telenga, 1934

Recorded from: Vayssières et al. 2001

Host information: *Liriomyza* spp. (Diptera, Agromyzidae) (Vayssières et al. 2001)

*Dacnusa* sp.

Recorded from: Vayssières et al. 2001

Host information: Unknown, but *Dacnusa* spp. are parasitoids of *Liriomyza* spp. (Diptera, Agromyzidae) (Vayssières et al. 2001)

**Genus: *Dinotrema*** Förster, 1863

*Dinotrema candidiapex* Fischer, 2014

Recorded from: Fischer 2014

Host information: Unknown

**Genus: *Synaldis*** Förster, 1863

*Synaldis dugainensis* Fischer, 2014

Recorded from: Fischer 2014

Host information: Unknown

*Synaldis robusticeps* Fischer, 2014

Recorded from: Fischer 2014

Host information: Unknown

##### Subfamily: Aphidiinae

**Genus: *Aphidius*** Nees, 1818

*Aphidius camerunensis* Mackauer, 1966

Recorded from: Stary et al. 1994

Host information: *Sitobion* spp. (Hemiptera, Aphididae) (Todorov 2009)

*Aphidius colemani* Viereck, 1912

Recorded from: Vayssières et al. 2001

Host information: Hemiptera, Aphididae, Genera: *Aphis*, *Aulacorthum*, *Brachycaudus*, *Brevicorinae*, *Capitophorus*, *Dysaphis*, *Hialopterus*, *Hayhurstia*, *Hyadaphis*, *Hypermizus*, *Macrosiphon*, *Melanaphis*, *Micromizus*, *Mizodes*, *Mizus*, *Pterocoma*, *Rhopalosiphum*, *Schizaphis*, *Thelaxis*, *Toxoptera* (Todorov 2009)

*Aphidius rosae* Haliday, 1833

Recorded from: Starý et al. 1977

Host information: *Macrosiphum rosae*, *Sitobion fragariae* (Hemiptera, Aphididae) (Mackauer and Stary 1967, Stary 1973)

*Aphidius seyrigi* Granger, 1949

Recorded from: Benoit 1957

Host information: Hemiptera, Aphididae

**Genus: *Diaeretiella*** Starý, 1960

*Diaeretiella rapae* (McIntosh, 1855)

Recorded from: Quilici et al. 1988

Host information: *Myzus persicae*, *Brevicoryne brassicae*, *Lipaphis erysimi* on cabbage, cauliflower, mustard, etc. *Aphis craccivora*, *Aphis fabae*, *Aphis gossypii*, *Brachycaudus helichrysi*, *Hysteroneura setariae*, and *Schizaphis graminum* (Hemiptera, Aphididae) (Raychaudhuri 1990)

**Genus: *Praon*** Haliday, 1833

*Praon* sp.

Recorded from: Vayssières et al. 2001

Host information: *Aphis* sp. (Vayssières et al. 2001) *Uroleucon sonchi*, *Hyperomyzus lactucae*, *Nasonovia ribisnigri*, *Macrosiphum euphorbiae* (Madl and van Achterberg 2014) (Hemiptera, Aphididae)

##### Subfamily: Braconinae

**Genus: *Bracon*** Fabricius, 1804

Bracon (Habrobracon) hebetor (Say, 1836)

Recorded from: Quilici et al. 2003

Host information: *Maruca vitrata* or *Maruca testulalis* (Lepidoptera, Crambidae), *Lampides boeticus* (Lepidoptera, Lycaenidae), *Corcyra cephalonica*, *Etiella zinckenella* (Lepidoptera, Pyralidae) (Madl and van Achterberg 2014)

**Genus: *Iphiaulax*** Förster, 1863

*Iphiaulax didymus* (Brullé, 1846)

Recorded from: Brullé 1846

Host information: Unknown

##### Subfamily: Charmontinae

**Genus: *Charmon*** Haliday, 1833

*Charmon ramagei* Rousse, 2013

Recorded from: Rousse 2013

Host information: Unknown

##### Subfamily: Cheloninae

**Genus: *Chelonus*** Panzer, 1806

Chelonus (Microchelonus) curvimaculatus Cameron, 1906

Recorded from: Quilici et al. 2003, Braet et al. 2012

Host information: *Nephopterix beharella* (Lepidoptera, Pyralidae) (Quilici et al. 2003), *Pectinophora gossypiella* (Lepidoptera, Gelechiidae), *Trichoplusia ni* (Lepidoptera, Notcuidae) (Chiri and Legner 1982, Jones 1986)

Chelonus (Microchelonus) matilei Braet & Rousse, 2012

Recorded from: Braet et al. 2012

Host information: Unknown

Chelonus (Chelonus) mayi Braet & Rousse, 2012

Recorded from: Braet et al. 2012

Host information: Unknown

Chelonus (Microchelonus) merdicus Rousse & Braet, 2012

Recorded from: Braet et al. 2012

Host information: Unknown

*Chelonus* sp.

Recorded from: Quilici et al. 2003

Host information: Unknown

##### Subfamily: Doryctinae

**Genus: *Ipodoryctes*** Granger, 1949

*Ipodoryctes* sp.

*Recorded from*: Zaldivar-Riveron et al. 2008

Host information: Parasitoid of xylophagus and stem-boring Coleoptera (Beyarslan 2015)

**Genus: *Rinamba*** Cameron, 1912

*Rinamba opacicollis* Cameron, 1912

Recorded from: Braet 2001

Host information: Parasitoid of xylophagus and stem-boring Coleoptera larvae (Beyarslan 2015)

##### Subfamily: Euphorinae

**Genus: *Centistes*** Haliday, 1835

*Centistes caloupile* Rousse & Braet, 2012

Recorded from: Rousse and Braet 2012

Host information: Unknown

**Genus: *Chrysopophthorus*** Goidanich, 1948

*Chrysopophthorus* sp.

Recorded from: Semeria and Quilici 1986

Host information: Unknown, but *Chrysopophtorus* spp. are endoparasitoidss of Chrysopidae (Neuroptera) (Séméria 1976)

**Genus: *Cosmophorus*** Ratzeburg, 1848

*Cosmophorus merdiculatus* Rousse & Braet, 2012

Recorded from: Rousse and Braet 2012

Host information: Unknown, but *Cosmophorus* spp. are endoparasitoidss of adult bark beetles (Coleoptera, Scolytinae) (Watanabe 1968)

**Genus: *Leiophron*** Nees, 1819

*Leiophron sarahae* Rousse & Braet, 2012

Recorded from: Rousse and Braet 2012

Host information: Unknown, but *Leiophron* spp. are parasitoids of Miridae and Psocoptera (Simbolotti et al. 2002)

*Leiophron yaeli* Rousse & Braet, 2012

Recorded from: Rousse and Braet 2012

Host information: Unknown, but *Leiophron* spp. are parasitoids of Miridae and Psocoptera (Simbolotti et al. 2002)

**Genus: *Meteorus*** Haliday, 1835

*Meteorus anthracnemis* de Saeger, 1946

Recorded from: Rousse and Braet 2012

Host information: Unknown, but *Meteorus* spp. are endoparasitoidss of Lepidoptera or Coleoptera (van Achterberg 1979; Zitani et al. 1997)

*Meteorus comonile* Rousse & Braet, 2012

Recorded from: Rousse and Braet 2012

Host information: Unknown, but *Meteorus* spp. are endoparasitoidss of Lepidoptera or Coleoptera (van Achterberg 1979; Zitani et al. 1997)

*Meteorus pulchricornis* Wesmael, 1835

Recorded from: Rousse and Braet 2012

Host information: Lepidoptera: Geometridae, Lasiocampidae, Lycaenidae, Lymantriidae, Noctuidae, Nolidae, Nymphalidae, (Huddleston 1980) Arctiidae, Papilionidae, (Maetô 1990) Plutellidae, (Okada 1989) and Tortricidae, (Berry 1997)

**Genus: *Syntretus*** Förster, 1863

*Syntretus massale* Rousse and Braet, 2012

Recorded from: Rousse and Braet 2012

Host information: Unknown, but *Syntretus* spp. are solitary endoparasitoidss of adult Hymenoptera (Ichneumonidae and Apidae (Rousse and Braet 2012)

##### Subfamily: Microgastrinae

**Genus: *Apanteles*** Förster, 1863

Apanteles aff. acutissimus Granger, 1949

Recorded from: Vayssières et al. 2001

Host information: *Diaphana indica* (Lepidoptera, Crambidae) (Vayssières et al. 2001)

*Apanteles bordagei* Giard, 1902

Recorded from: Rousse and Gupta 2013

Host information: Endoparasitoid of *Crobylophora daricella*, *Leucoptera caffeina*, *Leucoptera mayricki* (Lepidoptera, Lyonetiidae) (Ferrière 1936, Rousse and Gupta 2013)

*Apanteles fontinalis* de Saeger, 1944

Recorded from: Rousse and Gupta 2013

Host information: Unknown, but *Apanteles* are endoparasitoids of Lepidotera larvae (Farahani et al. 2012)

*Apanteles galleriae* Wilkinson, 1932

Recorded from: Rousse and Gupta 2013

Host information: *Achroia grisella*, *Galleria mellonella* and *Vitula edmansii* (Lepidoptera, Pyralidae) (Rousse and Gupta 2013)

Apanteles (Apanteles) minatchy Rousse & Gupta, 2013

Recorded from: Rousse and Gupta 2013

Host information: Unknown, but *Apanteles* are endoparasitoids of Lepidotera larvae (Farahani et al. 2012)

Apanteles (Apanteles) nigrofemoratus Granger, 1949

Recorded from: Rousse and Gupta 2013

Host information: *Tortyra* sp. (Lepidoptera, Choreutidae) (Rousse and Gupta 2013)

Apanteles (Apanteles) phasmina Rousse, 2013

Recorded from: Rousse and Gupta 2013

Host information: Unknown, but *Apanteles* spp. are endoparasitoids of Lepidotera larvae (Farahani et al. 2012)

Apanteles (Apanteles) romei Rousse, 2013

Recorded from: Rousse and Gupta 2013

Host information: *Diaphina* sp. (Lepidoptera, Pyralidae) on *Momordicha charantia* (Cucurbitaceae) (Rousse and Gupta 2013)

**Genus: *Cotesia*** Cameron, 1891

*Cotesia flavipes* Cameron, 1891

Recorded from: Kfir et al. 2002

Host information: About 30 lepidopteran species in Arctiidae, Brachodidae, Lymantriidae, Noctuidae, Pyralidae and Tortricidae, including major pests (Rousse and Gupta 2013)

*Cotesia plutellae* (Kurdumov, 1912)

Recorded from: Rousse and Gupta 2013

Host information: About 30 lepidopteran species in Arctiidae, Lasiocampidae, Lymantriidae, Noctuidae, Notodontidae, Nymphalidae, Pieridae, Plutellidae, Pterophoridae, Pyralidae and Tortricidae, including major pests (Rousse and Gupta 2013)

*Cotesia ruficrus* (Haliday, 1834)

Recorded from: Rousse and Gupta 2013

Host information: About 100 lepidopteran species in Arctiidae, Brachodidae, Dilobidae, Gelechiidae, Geometridae, Hesperiidae, Lasiocampidae, Lycaenidae, Lymantriidae, Noctuidae, Nymphalidae, Pieridae, Plutellidae, Pyralidae and Tortricidae (Rousse and Gupta 2013)

*Cotesia sesamiae* (Cameron, 1906)

Recorded from: Van Achterberg and Polaszek 1996

Host information: 15 species of Noctuidae and Pyralidae, including major pests (Rousse and Gupta 2013)

*Cotesia xavieri* Rousse, 2013

Recorded from: Rousse and Gupta 2013

Host information: Unknown, but *Apanteles* spp. are endoparasitoids of Lepidotera larvae (Rousse and Gupta 2013)

**Genus: *Diolcogaster*** Ashmead, 1900

*Diolcogaster austrina* (Wilkinson, 1929)

Recorded from: Aubert 1966

Host information: Unknown, but *Diolcogaster* spp. are endoparasitoids in 11 families of Lepidoptera including: Arctiidae, Geometridae, Lasiocampidae, Limacodidae, Lymantriidae, Noctuidae, Notodontidae, Plutellidae, Pyralidae, Tenthredinidae, and Thaumetopoeidae (Zeng et al. 2011)

*Diolcogaster curticornis* (Granger, 1949)

Recorded from: Rousse and Gupta 2013

Host information: *Brenthia leptocosma*, (Lepidoptera, Choreutidae) *Macalla* sp. (Lepidoptera, Pyralidae) (Rousse and Gupta 2013)

**Genus: *Distatrix*** Mason, 1981

*Distatrix belliger* (Wilkinson, 1929)

Recorded from: Rousse and Gupta 2013

Host information: *Helicoverpa armigera*, *Pseudaletia unipunctata* and *Mythimna* sp. (Lepidoptera, Noctuidae) (Rousse and Gupta 2013)

*Distatrix yunae* Rousse & Gupta, 2013

Recorded from: Rousse and Gupta 2013

Host information: Unknown, but *Distatrix* spp. are endoparasitoids of Lepidoptera larvae (Rousse and Gupta 2013)

**Genus: *Dodogaster*** Rousse, 2013

*Dodogaster grangeri* Rousse, 2013

Recorded from: Rousse and Gupta 2013

Host information: Unknown

**Genus: *Dolichogenidea*** Viereck, 1911

*Dolichogenidea ashoka* Rousse, 2013

Recorded from: Rousse and Gupta 2013

Host information: Unknown, but *Dolichogenidea* spp. are endoparasitoids of Lepidoptera larvae (Rousse and Gupta 2013)

*Dolichogenidea broadi* Rousse, 2013

Recorded from: Rousse and Gupta 2013

Host information: Unknown, but *Dolichogenidea* spp. are endoparasitoids of Lepidoptera larvae (Rousse and Gupta 2013)

*Dolichogenidea lumba* Rousse & Gupta, 2013

Recorded from: Rousse and Gupta 2013

Host information: Unknown, but *Dolichogenidea* spp. are endoparasitoids of Lepidoptera larvae (Rousse and Gupta 2013)

*Dolichogenidea uru* Rousse & Gupta, 2013

Recorded from: Rousse and Gupta 2013

Host information: Unknown, but *Dolichogenidea* spp. are endoparasitoids of Lepidoptera larvae (Rousse and Gupta 2013)

*Dolichogenidea villemantae* Rousse, 2013

Recorded from: Rousse and Gupta 2013

Host information: Unknown, but *Dolichogenidea* spp. are endoparasitoids of Lepidoptera larvae (Rousse and Gupta 2013)

**Genus: *Exoryza*** Mason, 1981

*Exoryza safranum* Rousse & Gupta, 2013

Recorded from: Rousse and Gupta 2013

Host information: Unknown, but *Exoryza* spp. are endoparasitoids of Lepidoptera larvae (Rousse and Gupta 2013)

**Genus: *Glyptapanteles*** Ashmead, 1904

*Glyoptapanteles antsirabensis* (Granger, 1949)

Recorded from: Rousse and Gupta 2013

Host information: Unknown, but *Glyptapanteles* spp. are endoparasitoids of Lepidoptera larvae (Rousse and Gupta 2013)

*Glyptapanteles chidra* Rousse & Gupta, 2013

Recorded from: Rousse and Gupta 2013

Host information: Unknown, but *Glyptapanteles* spp. are endoparasitoids of Lepidoptera larvae (Rousse and Gupta 2013)

*Glyptapanteles ficus* (Granger, 1949)

Recorded from: Rousse and Gupta 2013

Host information: *Naroma signifera* (Lepidoptera, Lymantriidae) (Rousse and Gupta 2013)

*Glyptapanteles subandinus* (Blanchard, 1947)

Recorded from: Rousse and Gupta 2013

Host information: *Achyra bifidalis* (Lepidoptera, Pyralidae), *Phthorimaea opercullela* and *Scrobipalpula absoluta* (Lepidoptera, Gelechiidae) on *Solanum tuberosum* (Solanaceae) (Rousse and Gupta 2013)

**Genus: *Microplitis*** Förster, 1863

*Microplitis subsulcatus* Granger, 1949

Recorded from: Rousse and Gupta 2013

Host information: Unknown, but *Microplitis* spp. are endoparasitoids of Lepidoptera larvae (Rousse and Gupta 2013)

**Genus: *Nyereria*** Mason, 1981

*Nyereria ganges* Rousse & Gupta, 2013

Recorded from: Rousse and Gupta 2013

Host information: Unknown, but *Nyereria* spp. are endoparasitoids of macrolepidoptera larvae (Rousse and Gupta 2013)

*Nyereria mayurus* Rousse & Gupta, 2013

Recorded from: Rousse and Gupta 2013

Host information: Unknown, but *Nyereria* spp.are endoparasitoids of macrolepidoptera larvae (Rousse and Gupta 2013)

**Genus: *Parapanteles*** Ashmead, 1900

*Parapanteles covino* Rousse & Gupta, 2013

Recorded from: Rousse and Gupta 2013

Host information: Unknown, but *Parapanteles* spp. are endoparasitoids in 7 families of Lepidoptera including: Arctiidae, Noctuidae, Notodontidae, Elachistidae, Saturniidae, Geometridae, Gelechiidae (Valerio et al. 2009)

*Parapanteles darignac* Rousse & Gupta, 2013

Recorded from: Rousse and Gupta 2013

Host information: Unknown. but *Parapanteles* spp. are endoparasitoids in 7 families of Lepidoptera including: Arctiidae, Noctuidae, Notodontidae, Elachistidae, Saturniidae, Geometridae, Gelechiidae (Valerio et al. 2009)

**Genus: *Venanides*** Mason, 1981

*Venanides curticornis* (Granger, 1949)

Recorded from: Rousse and Gupta 2013

Host information: Unknown, but *Venanides* spp. are endoparasitoids of microlepidoptera larvae (Rousse and Gupta 2013)

**Genus: *Wilkinsonellus*** Mason, 1981

*Wilkinsonellus narangahus* Rousse & Gupta, 2013

Recorded from: Rousse and Gupta 2013

Host information: Unknown

##### Subfamily: Gnamptodontinae

**Genus: *Gnamptodon*** Haliday, 1833

*Gnamptodon legorskyi* Fischer, 2014

Recorded from: Fischer 2014

Host information: Unknown, but *Gnamptodon* spp. are parasitoids of Nepticulidae.

##### Subfamily: Opiinae

**Genus: *Diachasmimorpha*** Viereck, 1913

*Diachasmimorpha fullawayi* (Silvestri, 1913)

Recorded from: Quilici et al. 2003

Host information: *Ceratitis capitata* (Diptera, Tephritidae) (Quilici et al. 2003, Fischer and Madl 2008)

*Diachasmimorpha tryoni* (Cameron, 1911)

Recorded from: Hurtrel et al. 1999

Host information: At least 6 species of Tephritidae (Diptera) (Ramadan et al. 1994, Wharton and Gilstrap 1983)

**Genus: *Fopius*** Wharton, 1987

*Fopius arisanus* (Sonan, 1932)

Recorded from: Quilici et al. 2003

Host information: *Bactrocera zonata*, *Ceratitis capitata*, *Ceratitis catoirii*, *Ceratitis rosa*, *Dacus ciliatus*, *Dacus demmerezi*, *Neoceratitis cyanescens* (Diptera, Tephritidae) (Fischer and Madl 2008)

**Genus: *Opius*** Wesmael, 1835

*Opius dissitus* Muesebeck, 1963

Recorded from: Vayssières et al. 2001

Host information: *Liriomyza* spp. (Diptera, Agromyzidae) (Stegmaier 1972, Schuster and Wharton 1993, Vayssières et al. 2001)

Opius (Utetes) coriacitergum Fischer, 2014

Recorded from: Fischer 2014

Host information: Unknown, but Opiinae are parasitoids of Cyclorrhapha (Diptera) larvae (Ameri et al. 2014)

Opius (Tolbia) karlmayi Fischer, 2014

Recorded from: Fischer 2014

Host information: Unknown, but Opiinae are parasitoids of Cyclorrhapha (Diptera) larvae (Ameri et al. 2014)

Opius (Utetes) lareunionensis Fischer, 2014

Recorded from: Fischer 2014

Host information: Unknown, but Opiinae are parasitoids of Cyclorrhapha (Diptera) larvae (Ameri et al. 2014)

Opius (Gastrosema) laticrenis Fischer, 2014

Recorded from: Fischer 2014

Host information: Unknown, but Opiinae are parasitoids of Cyclorrhapha (Diptera) larvae (Ameri et al. 2014)

Opius (Opius) raoiformis Fischer, 2014

Recorded from: Fischer 2014

Host information: Unknown, but Opiinae are parasitoids of Cyclorrhapha (Diptera) larvae (Ameri et al. 2014)

Opius (Utetes) semipilosus Fischer, 2014

Recorded from: Fischer 2014

Host information: Unknown, but Opiinae are parasitoids of Cyclorrhapha (Diptera) larvae (Ameri et al. 2014)

Opius (Gastrosema) waterloti Granger, 1949

Recorded from: Fischer and Madl 2008

Host information: Unknown, but Opiinae are parasitoids of Cyclorrhapha (Diptera) larvae (Ameri et al. 2014)

**Genus: *Psyttalia*** Walker, 1860

*Psyttalia cosyrae* (Wilkinson, 1927)

Recorded from: Doubtful record. Fischer (1987) recorded it from Reunion because “Réunion, Mayotte” was indicated on the label.

Host information: *Dacus* sp. (Diptera, Tephritidae) (Fischer and Madl 2008)

*Psyttalia distinguenda* (Granger, 1949)

*Recorded from*: Wharton et al. 1999

Host information: *Ceratitis capitata* (Diptera, Tephritidae) (Quilici et al. 2003, Fischer and Madl 2008)

*Psyttalia fletcheri* (Sylvestri, 1916)

Recorded from: Hurtrel et al. 1999

Host information: *Bactrocera cucurbitae*, *Ceratitis capitata* and *Dacus ciliatus* (Diptera, Tephritidae) (Fischer and Madl 2008)

*Psyttalia insignipennis* (Granger, 1949)

Recorded from: Wharton et al. 1999

Host information: *Ceratitis capitata*, *Ceratitis catoirii* and *Neoceratitis cyanescens* (Diptera, Tephritidae) (Quilici et al. 2003, Fischer and Madl 2008)

*Psyttalia phaeostigma* (Wilkinson, 1927)

Recorded from: Fischer 1987

Host information: *Dacus ciliatus* and *Dacus demmerezi* (Diptera, Tephritidae) (Fischer and Madl 2008)

*Psyttalia sanctamarina* (Fischer, 1980)

Recorded from: Fischer 1980

Host information: *Spathulina aroleuca* (Diptera, Tephritidae) (Fischer and Madl 2008)

*Psyttalia subsulcata* (Granger, 1949)

Recorded from: Fischer and Madl 2008

Host information: *Spathulina aroleuca* (Diptera, Tephritidae) (Fischer and Madl 2008)

#### Family: ICHNEUMONIDAE

##### Subfamily: Banchinae

**Genus: *Himertosoma*** Schimedeknecht, 1900

*Himertosoma bebourensis* (Benoit, 1957)

Recorded from: Benoit 1957

Host information: Unknown, but Banchinae are parasitoid of Lepidoptera larvae (Rousse and Villemant 2012)

*Himertosoma striata* (Seyrig, 1932)

Recorded from: Rousse and Villemant 2012

Host information: Unknown, but Banchinae are parasitoid of Lepidoptera larvae (Rousse and Villemant 2012)

##### Subfamily: Campopleginae

**Genus: *Campoplex*** Gravenhorst, 1829

*Campoplex techer* Rousse & Villemant, 2012

Recorded from: Rousse and Villemant 2012

Host information: Unknown, but Campopleginae are generally parasitoids of Lepidoptera and Symphyta but they sometimes parasitize Coleoptera and Neuroptera (Rousse and Villemant 2012)

**Genus: *Diadegma*** Förster, 1869

*Diadegma insulare* (Cresson, 1865)

Recorded from: Rousse and Villemant 2012

Host information: *Plutella xylostella*, *Plutella opercula*, *Plutella omissa*, *Plutella armoraciae* (Lepidoptera, Plutellidae) and *Hellula undalis* (Lepidoptera, Pyralidae) (Sourakov and Mitchell 2000, Rousse and Villemant 2012)

*Diadegma mollipla* (Holmgren, 1868)

Recorded from: Vayssières et al. 2001

Host information: *Plutella xylostella*, (Lepidoptera, Plutellidae) and *Phthorimaea operculella* (Lepidoptera, Gelechiidae) (Rousse and Villemant 2012)

**Genus: *Dusona*** Cameron, 1901

*Dusona douraguia* Rousse & Villemant, 2012

Recorded from: Rousse and Villemant 2012

Host information: Unknown, but Campopleginae are generally parasitoids of Lepidoptera and Symphyta but they sometimes parasitize Coleoptera and Neuroptera (Rousse and Villemant 2012)

*Dusona pauliani* (Benoit, 1957)

Recorded from: Rousse and Villemant 2012

Host information: Unknown, but Campopleginae are generally parasitoids of Lepidoptera and Symphyta but they sometimes parasitize Coleoptera and Neuroptera (Rousse and Villemant 2012)

**Genus: *Enytus*** Cameron, 1905

*Enytus huet* Rousse & Villemant, 2012

Recorded from: Rousse and Villemant 2012

Host information: Unknown, but Campopleginae are generally parasitoids of Lepidoptera and Symphyta but they sometimes parasitize Coleoptera and Neuroptera (Rousse and Villemant 2012)

**Genus: *Eriborus*** Förster, 1869

*Eriborus cadjee* Rousse & Villemant, 2012

Recorded from: Rousse and Villemant 2012

Host information: Unknown, but Campopleginae are generally parasitoids of Lepidoptera and Symphyta but they sometimes parasitize Coleoptera and Neuroptera (Rousse and Villemant 2012)

*Eriborus pallipes* (Brullé, 1846)

Recorded from: Rousse and Villemant 2012

Host information: Unknown, but Campopleginae are generally parasitoids of Lepidoptera and Symphyta but they sometimes parasitize Coleoptera and Neuroptera (Rousse and Villemant 2012)

**Genus: *Hyposoter*** Förster 1869

*Hyposoter reunionis* (Benoit, 1957)

Recorded from: Benoit 1957

Host information: Unknown, but Campopleginae are generally parasitoids of Lepidoptera and Symphyta but they sometimes parasitize Coleoptera and Neuroptera (Rousse and Villemant 2012)

**Genus: *Venturia*** Schrottky, 1902

*Venturia canescens* (Gravenhorst, 1829)

Recorded from: Rousse and Villemant 2012

Host information: 23 species belonging to the families Pyralidae, Noctuidae, Tortricidae, Gelechiidae, Tineidae and Yponomeutidae (Lepidoptera) (Rousse and Villemant 2012)

**Genus: *Xanthocampoplex*** Morley, 1913

*Xanthocampoplex huberti* Rousse & Villemant, 2012

Recorded from: Rousse and Villemant 2012

Host information: Unknown, but Campopleginae are generally parasitoids of Lepidoptera and Symphyta but they sometimes parasitize Coleoptera and Neuroptera (Rousse and Villemant 2012)

##### Subfamily: Cremastinae

**Genus: *Pristomerus*** Curtis, 1836

*Pristomerus rivier* Rousse & Villemant, 2012

Recorded from: Rousse and Villemant 2012

Host information: *Plutella xylostella* (Plutellidae) (Rousse and Villemant 2012) and *Prophantis smaragdina* (Chartier et al. 2013, Identified by Rousse 2012 unpublished data)

**Genus: *Temelucha*** Meyrick, 1909

*Temelucha basiornata* (Cameron, 1911)

Recorded from: Rousse and Villemant 2012

Host information: Unidentified rice stem borer, probably *Diopsis* sp. (Diptera, Diopsidae) (Zwart 1998)

*Temelucha labusi* Rousse & Villemant, 2012

Recorded from: Rousse and Villemant 2012

Host information: Unknown, but Cremastinae are endoparasitoids of Lepidoptera or Coleoptera larvae in leave folds, tunnels, buds, galls or other concealed situations (Rousse and Villemant 2012)

*Temelucha minuta* (Morley, 1912)

Recorded from: Rousse and Villemant 2012

Host information: *Grapholita critica* (Lepidoptera, Tortricidae), *Etiella behrii* (Lepidoptera, Pyralidae), *Bilobata subsecivella* (Lepidoptera, Gelechiidae) on peanut, *Phthorimaea operculella* (Lepidoptera, Gelechiidae) on potato (Gauld 1980, Rousse and Villemant 2012)

*Temelucha pestifer* (Morley, 1913)

Recorded from: Benoit 1957

Host information: An unidentified stem borer on *Oryza sativa* (Poaceae) field (Rousse and Villemant 2012)

*Temelucha talibarti* Rousse, Villemant & Seyrig, 2011

Recorded from: Rousse and Villemant 2012

Host information: Unknown, but Cremastinae are endoparasitoids of Lepidoptera or Coleoptera larvae in leave folds, tunnels, buds, galls or other concealed situations (Rousse and Villemant 2012)

*Temelucha* sp. 1

Recorded from: Rousse and Villemant 2012

Host information: Unknown, but Cremastinae are endoparasitoids of Lepidoptera or Coleoptera larvae in leave folds, tunnels, buds, galls or other concealed situations (Rousse and Villemant 2012)

*Temelucha* sp. 2

Recorded from: Rousse and Villemant 2012

Host information: Unknown, but Cremastinae are endoparasitoids of Lepidoptera or Coleoptera larvae in leave folds, tunnels, buds, galls or other concealed situations (Rousse and Villemant 2012)

**Genus: *Trathala*** Cameron, 1899

*Trathala annulicornis* (Tosquinet, 1896)

Recorded from: Rousse and Villemant 2012

Host information: Unknown, but Cremastinae are endoparasitoids of Lepidoptera or Coleoptera larvae in leave folds, tunnels, buds, galls or other concealed situations (Rousse and Villemant 2012)

*Trathala flavoorbitalis* (Cameron, 1907)

Recorded from: Rousse and Villemant 2012

Host information: Seventy-eight host records, all Lepidoptera (Gelechoidea, Noctuoidea, Pyraloidea, Tineoidea and Tortricoidea) (Rousse and Villemant 2012)

##### Subfamily: Cryptinae

**Genus: *Acrolyta*** Förster, 1868

*Acrolyta dindar* Rousse & Villemant, 2012

Recorded from: Rousse and Villemant 2012

Host information: Unknown, but Cryptinae generally are ectoparasitoids of Holometabola pupae or prepupae. Some species are endoparasitoids, and some species parasitize the egg masses of Araneae and Pseudoscorpionida. (Rousse and Villemant 2012)

**Genus: *Diatora*** Förster, 1868

*Diatora albiscapus* (Seyrig, 1952)

Recorded from: Rousse and Villemant 2012

Host information: *Apanteles* sp. (Hymenoptera, Braconidae) ex *Napta serratilinea* (Lepidoptera, Lasiocampidae) and *Nephele densoi* (Lepidoptera, Sphingidae) on mimosa (*Mimosa* sp.) (Seyrig 1952, Rousse and Villemant 2012)

**Genus: *Paraphylax*** Förster, 1869

*Paraphylax mussar* Rousse & Villemant, 2012

Recorded from: Rousse and Villemant 2012

Host information: Unknown, but *Paraphylax* spp. are parasitoids of *Heliothis armigera* (Lepidoptera, Noctuidae) (Choudhary et al. 1983)

*Paraphylax transversatoria* (Seyrig, 1952)

Recorded from: Rousse and Villemant 2012

Host information: Unknown, but *Paraphylax* spp. are parasitoids of *Heliothis armigera* (Lepidoptera, Noctuidae) (Choudhary et al. 1983)

**Genus: *Phygadeuon*** Gravenhorst, 1829

*Phygadeuon nativel* Rousse & Villemant, 2012

Recorded from: Rousse and Villemant 2012

Host information: Unknown, but *Phygadeuon* spp. are parasitoids of *Musca domestica* (Diptera, Muscidae) and *Rhagoletis cerasi* (Diptera, Tephritidae) (Rousse and Villemant 2012)

##### Subfamily: Diplazontinae

**Genus: *Diplazon*** Nees, 1918

*Diplazon laetatorius* (Fabricius, 1781)

Recorded from: de Saussure 1892

Host information: Parasitoid of a wide range of Diptera, especially aphidophagous syrphid flies (more than 50 host species reported in Syrphidae) (Rousse and Villemant 2012)

##### Subfamily: Ichneumoninae

**Genus: *Crytea*** Cameron, 1906

*Crytea albitrochanterata* (Heinrich, 1938)

Recorded from: Rousse and Villemant 2012

Host information: Unknown, but Ichneumoninae are endoparasitoids of Lepidoptera larvae or pupae (Rousse and Villemant 2012)

**Genus: *Ichneumon*** Fabricius, 1798

*Ichneumon unicinctus* Brullé, 1846

Recorded from: Rousse and Villemant 2012

Host information: *Charadrina* sp., *Sciomesia biluma*, *Sesamia calamistis* (Lepidoptera, Noctuidae, Amphipyrinae) (Zwart 1998)

##### Subfamily: Mesochorinae

**Genus: *Mesochorus*** Gravenhorst, 1829

*Mesochorus cariniferus* Benoit, 1955

Recorded from: Rousse and Villemant 2012

Host information: Unknown, but Mesochorinae are hyperparasitoids of Ichneumonoidea (Hymenoptera) or Tachinidae (Diptera) (Rousse and Villemant 2012)

*Mesochorus* sp.

Recorded from: Rousse and Villemant 2012

Host information: Unknown, but Mesochorinae are hyperparasitoids of Ichneumonoidea (Hymenoptera) or Tachinidae (Diptera) (Rousse and Villemant 2012)

##### Subfamily: Metopiinae

**Genus: *Triclistus*** Förster, 1869

*Triclistus aitkeni* (Cameron, 1897)

Recorded from: Rousse and Villemant 2012

Host information: *Cnaphalocrocis medinalis* (Guenée) (Lepidoptera: Pyralidae) on rice (*Oryza sativa*) (Rousse and Villemant 2012)

##### Subfamily: Ophioninae

**Genus: *Enicospilus*** Stephens, 1835

*Enicospilus angustatus* (Brullé, 1846)

Recorded from: Bordage 1914

Host information: Unknown, but *Enicospilus* spp. are nocturnal endoparasitoids of Lepidoptera (Rousse and Villemant 2012)

*Enicospilus betanimenus* (Saussure, 1892)

Recorded from: Rousse and Villemant 2012

Host information: *Achaea faber*, *Dysgonia pudica*, *Dysgonia triplocyma*, *Heliophisma klugii*, and *Tatorinia rufipennis* (Lepidoptera, Noctuidae) (Rousse and Villemant 2012)

*Enicospilus dolosus* (Tosquinet, 1896)

Recorded from: Rousse and Villemant 2012

Host information: *Anomis leona* (Lepidoptera, Noctuidae) and *Haritalodes derogate* (Lepidoptera, Crambidae) (Rousse and Villemant 2012)

*Enicospilus dubius* (Tosquinet, 1896)

Recorded from: Rousse and Villemant 2012

Host information: *Anomis leona*, *Chrysodeixis chalcites* (Lepidoptera, Noctuidae) and *Plusia* sp. (Lepidoptera, Plusiidae) (Gauld and Mitchell 1978)

*Eniscopilus grandiflavus* Townes, 1973

Recorded from: Rousse and Villemant 2012

Host information: Unknown, but *Enicospilus* spp. are nocturnal endoparasitoids of Lepidoptera (Rousse and Villemant 2012)

*Enicospilus helvolus* Gauld & Mitchell, 1978

Recorded from: Rousse and Villemant 2012

Host information: Unknown, but *Enicospilus* spp. are nocturnal endoparasitoids of Lepidoptera (Rousse and Villemant 2012)

*Enicospilus hova* Gauld & Mitchell, 1978

Recorded from: Gauld and Mitchell 1978

Host information: Unknown, but *Enicospilus* spp. are nocturnal endoparasitoids of Lepidoptera (Rousse and Villemant 2012)

*Enicospilus mauritii* (de Saussure, 1892)

Recorded from: Bordage 1914

Host information: *Chilo sacchariphagus* (Lepidoptera, Pyralidae), *Leucania loreyi* (Lepidoptera, Noctuidae, Hadeninae), *Mythimna loreyi* (Lepidoptera, Noctuidae), *Olethreutes schistaceanus*, *Tetramoera schistaceana* (Lepidoptera, Tortricidae, Olethreutinae), *Procherata sacchariphaga*, *Procerata venostata* (Lepidoptera, Brachodidae), *Sesamia calamistis*, *Sesamia inferens*, *Sesamia nonagrioides*, *Speia vuteria* (Lepidoptera, Noctuidae, Amphipyrinae) (Rousse and Villemant 2012)

*Enicospilus rufus* (Brullé, 1846)

Recorded from: Benoit 1957

Host information: *Ctenoplusia limbirena*, *Trigonodes hyppasia* (Lepidoptera, Noctuidae) (Rousse and Villemant 2012).

*Enicospilus ruscus* Gauld & Mitchell, 1978

Recorded from: Rousse and Villemant 2012

Host information: *Mythimna loreyi*, *Sesamia botanephaga*, *Sesamia veturia*, (Lepidoptera, Noctuidae) (Rousse and Villemant 2012)

*Enicospilus sesamiae* Delobel, 1974

Recorded from: Zwart 1998

Host information: *Procerata venosata* (Lepidoptera, Brachodidae) (Gauld and Mitchell 1978), *Busseola fusca*, *Sesamia calamistis* and *Sesamia vuteria* (Lepidoptera, Noctuidae) (Zwart 1998)

*Enicospilus transvaalensis* Cameron, 1911

Recorded from: Rousse and Villemant 2012

Host information: *Mythimna* sp. and *Sesamia calamistis* (Lepidoptera, Noctuidae) (Rousse and Villemant 2012)

*Enicospilus vitry* Rousse & Villemant, 2012

Recorded from: Rousse and Villemant 2012

Host information: Unknown, but *Enicospilus* spp. are nocturnal endoparasitoids of Lepidoptera (Rousse and Villemant 2012)

##### Subfamily: Orthocentrinae

**Genus: *Megastylus*** Schiødte, 1838

*Megastylus vagabundus* Seyrig, 1934

Recorded from: Rousse and Villemant 2012

Host information: Unknown, but Orthocentrinae are endoparasitoids of Mycetophilidae and Sciaridae (Diptera) (Rousse and Villemant 2012)

**Genus: *Orthocentrus*** Gravenhorst, 1829

*Orthocentrus urbanus* Seyrig, 1934

Recorded from: Rousse and Villemant 2012

Host information: Unknown, but Orthocentrinae are endoparasitoids of Mycetophilidae and Sciaridae (Diptera) (Rousse and Villemant 2012)

**Genus: *Pantisarthrus*** Förster, 1871

*Pantisarthrus isolatus* (Benoit, 1957)

Recorded from: Benoit 1957

Host information: Unknown, but Orthocentrinae are endoparasitoids of Mycetophilidae and Sciaridae (Diptera) (Rousse and Villemant 2012)

**Genus: *Proclitus*** Förster, 1869

*Proclitus ligatus* (Seyrig, 1934)

Recorded from: Rousse and Villemant 2012

Host information: Unknown, but Orthocentrinae are endoparasitoids of Mycetophilidae and Sciaridae (Diptera) (Rousse and Villemant 2012)

**Genus: *Stenomacrus*** Förster, 1869

*Stenomacrus payet* Rousse & Villemant, 2012

Recorded from: Rousse and Villemant 2012

Host information: Unknown, but Orthocentrinae are endoparasitoids of Mycetophilidae and Sciaridae (Diptera) (Rousse and Villemant 2012)

**Genus: *Tariqia*** Rousse and Villemant, 2012

*Tariqia stellaris* Rousse & Villemant, 2012

Recorded from: Rousse and Villemant 2012

Host information: Unknown, but Orthocentrinae are endoparasitoids of Mycetophilidae and Sciaridae (Diptera) (Rousse and Villemant 2012)

##### Subfamily: Pimplinae

**Genus: *Echthromorpha*** Holmgren, 1868

*Echthromorpha spinator* (Fabricius, 1798)

Recorded from: Rousse and Villemant 2012

Host information: *Nagia linteola* (Lepidoptera, Noctuidae) (Rousse and Villemant 2012)

**Genus: *Itoplectis*** (Förster, 1869)

*Itoplectis albipes* Seyrig 1932

Recorded from: Rousse and Villemant 2012

Host information: Unknown, but morphological studies indicate that *Itoplectis albipes* uses echolocation when foraging for hosts (Broad and Quicke 2000)

**Genus: *Xanthophenax*** Saussure, 1892

*Xanthophenax defector* Seyrig, 1932

Recorded from: Rousse and Villemant 2012

Host information: Unknown, but Pimplinae are generally ectoparasitoids of pupae and larvae of Holometabola. Some species parasitize adult and egg sacs od Araneae (Rousse and Villemant 2012)

*Xanthophenax pacificator* Seyrig, 1932

Recorded from: Rousse and Villemant 2012

Host information: Unknown, but Pimplinae are generally ectoparasitoids of pupae and larvae of Holometabola. Some species parasitize adult and egg sacs od Araneae (Rousse and Villemant 2012)

*Xanthophenax xanthomelas* (Brullé, 1846)

Recorded from: Rousse and Villemant 2012

Host information: Unknown, but Pimplinae are generally ectoparasitoids of pupae and larvae of Holometabola. Some species parasitize adult and egg sacs od Araneae (Rousse and Villemant 2012)

**Genus: *Xanthopimpla*** de Saussure, 1892

*Xanthopimpla stemmator* (Thunberg, 1824)

Recorded from: Kfir et al. 2002

Host information: More than 30 species of Pyralidae (Lepidoptera) (Rousse and Villemant 2012)

**Genus: *Zatypota*** Förster, 1869

*Zatypota inexpectata* (Seyrig, 1932)

Recorded from: Rousse and Villemant 2012

Host information: Unknown, but Pimplinae are generally ectoparasitoids of pupae and larvae of Holometabola. Some species parasitize adult and egg sacs od Araneae (Rousse and Villemant 2012)

##### Subfamily: Tersilochinae

**Genus: *Aneuclis*** Förster, 1869

*Aneuclis larga* Khalaim, 2009

Recorded from: Rousse and Villemant 2012

Host information: Unknown, but most Tersilochinae are endoparasitoids of Coleoptera (generally Curculionidae and Chrysomelidae). Some species parasitize Symphyta (Hymenoptera) (Rousse and Villemant 2012)

**Genus: *Diaparsis*** Förster, 1869

*Diaparsis ramassamy* Rousse & Villemant, 2012

Recorded from: Rousse and Villemant 2012

Host information: Unknown, but most Tersilochinae are endoparasitoids of Coleoptera (generally Curculionidae and Chrysomelidae). Some species parasitize Symphyta (Hymenoptera) (Rousse and Villemant 2012)

##### Subfamily: Tryphoninae

**Genus: *Netelia*** Gray, 1860

*Netelia melanopus* (Brullé, 1846)

Recorded from: de Saussure 1892

Host information: *Mythimna loreyi*, *Sesamia calamistis*, *Sesamia inferens* (Lepidoptera, Noctuidae), *Olethreutes schistaceanus* (Lepidoptera, Tortricidae) and *Chilo sacchariphagus* (Lepidoptera, Pyralidae) (Rousse and Villemant 2012)

*Netelia* sp.

Recorded from: Rousse and Villemant 2012

Host information: Unknown, but *Netelia* spp. are ectoparasitoids of Lepidoptera larvae (Rousse and Villemant 2012)

### Superfamily: CHALCIDOIDEA

#### Family: AGAONIDAE

##### Subfamily: Agaoninae

**Genus: *Elisabethiella*** Grandi, 1928

*Elisabethiella reflexa* Wiebes, 1975

Recorded from: Wiebes 1975

Host information: *Ficus reflexa* Thunb. (Moraceae) and *Ficus aldabrensis* Baker C.C. Berg (Wiebes 1975)

**Genus: *Nigeriella*** Wiebes, 1974

*Nigeriella avicola* Wiebes, 1975

Recorded from: Wiebes 1975

Host information: *Ficus rubra* Vahl (Moraceae) (Wiebes 1975)

**Genus: *Platyscapa*** Motschoulsky, 1863

*Platyscapa etiennei* Wiebes, 1977

Recorded from: Wiebes 1977

Host information: *Ficus salicifolia* Vahl (Moraceae) (Wiebes 1977)

##### Subfamily: Kradibiinae

**Genus: *Ceratosolen*** Mayr, 1885

*Ceratosolen coecus* (Coquerel, 1855)

Recorded from: Coquerel 1855

Host information: *Ficus mauritiana* Lam. (Moraceae) (Coquerel 1855)

**Genus: *Kradibia*** Saunders, 1883

*Kradibia etiennei* Wiebes, 1990

Recorded from: Wiebes and Gompton 1990

Host information: *Ficus lateriflora* Vahl (Moraceae) (Wiebes and Gompton 1990)

##### Subfamily: Sycophaginae

**Genus: *Sycophaga*** Westwood, 1840

*Sycophaga explorator* (Coquerel, 1855)

Recorded from: Coquerel 1855

Host information: Unknown. But *Sycophaga* are parasites of figs (Coquerel 1855)

#### Family: APHELINIDAE

##### Subfamily: Aphelininae

**Genus: *Aphelinus*** Dalman, 1820

*Aphelinus asychis* Walker, 1839

Recorded from: Vayssières et al. 2001

Host information: Hemiptera, Aphididae (Vayssières et al. 2001)

*Aphelinus gossypii* Timberlake, 1924

Recorded from: Quilici et al. 2003

Host information: Aphididae (Hemiptera) (Vayssières et al. 2001)

*Aphelinus mariscusae* (Risbec, 1957)

Recorded from: Risbec 1957

Host information: Aphididae (Hemiptera) and particularly Micromyzus (Kugegania) ageni (Polaszek and Hayat 1989)

*Aphelinus varipes* (Förster, 1841)

Recorded from: Vayssières et al. 2001

Host information: Aphididae (Hemiptera) (Vayssières et al. 2001)

**Genus: *Aphytis*** Howard, 1900

*Aphytis africanus* Quednau, 1964

Recorded from: Quilici et al. 2003

Host information: *Aonidiella aurantii* (Hemiptera, Diaspididae) (Triapitsyn and Kim 2008)

*Aphytis coheni* DeBach, 1960

Recorded from: Quilici et al. 2003

Host information: *Aspidiotus nerii* (Hemiptera, Diaspididae) (Triapitsyn and Kim 2008)

*Aphytis holoxanthus* DeBach, 1960

Recorded from: Quilici et al. 2003

Host information: *Chrysomphalus aonidum* (Hemiptera, Diaspididae) (Triapitsyn and Kim 2008)

*Aphytis lepidosaphes* Compere, 1955

Recorded from: Quilici et al. 2003

Host information: *Lepidosaphes beckii* (Hemiptera, Diaspididae) (Triapitsyn and Kim 2008)

*Aphytis lignanensis* Compere, 1955

Recorded from: Quilici et al. 2003

Host information: *Aonidiella aurantii*, *Pseudaulacaspis pentagona* (Hemiptera, Diaspididae) (Luck and Podoler 1985)

*Aphytis melinus* DeBach, 1959

Recorded from: Quilici et al. 2003

Host information: *Aonidiella aurantii* (Hemiptera, Diaspididae) (Luck and Podoler 1985)

**Genus: *Marietta*** Motschulsky, 1863

*Marietta leopardina* Motschulsky, 1863

Recorded from: Quilici et al. 2003

Host information: Hyperparasitoid of 48 hemiptera and scales parasitic hymenoptera species (Shabaan 2003)

##### Subfamily: Calesinae

**Genus: *Cales*** Howard, 1907

*Cales noacki* Howard, 1907

Recorded from: Etienne 1977

Host information: *Aleurothrixus floccosus*, (Miret and Garcia-Marì 2001) *Aleurotrachelus atratus* (Borowiec et al. 2007) (Hemiptera, Aleyrodidae)

##### Subfamily Coccophaginae

**Genus: *Coccophagus*** Westwood, 1833

*Coccophagus ceroplastae* (Howard, 1985)

Recorded from: Quilici et al. 2003

Host information: *Coccus viridis*, *Coccus hesperidum*, *Saissetia coffeae*, *Ceroplastes* spp., *Pulvinaria psidii*, *Pulvinaria polygonata* (Hemiptera, Coccoidea), scales and mealybugs belonging to Diaspididae and Pseudococcidae (Hemiptera) have also been recorded as hosts (Hayat 1998)

*Coccophagus cowperi* Girault, 1917

Recorded from: Quilici et al. 2003

Host information: *Saissetia oleae* (Clausen 1956), *Saissetia coffeae* (Shabaan 2005) (Hemiptera, Coccidae)

*Coccophagus rusti* Compere, 1928

Recorded from: Quilici et al. 2003

Host information: *Saissetia oleae* (Hemiptera, Coccidae) (Daane et al. 1991)

**Genus: *Encarsia*** Förster, 1878

*Encarsia acaudaleyrodis* Hayat, 1976

Recorded from: Vayssières et al. 2001

Host information: *Acaudaleyrodes rachipora*, *Tetraleurodes leguminicola*, *Bemisia tabaci* (Hemiptera, Aleyrodidae) (Ghahari et al. 2011)

*Encarsia brasiliensis* (Hempel, 1904)

Recorded from: Vayssières et al. 2001

Host information: *Aleurodicus dispersusBemisia tabaci*, *Lecanoideus floccissimusTrialeurodes vaporarium* (Homoptera, Aleyrodidae) (Magnet and Onillon 1997, Hernandez-Suarez et al. 2000)

*Encarsia citrina* (Craw, 1891)

Recorded from: Vayssières et al. 2001

Host information: Several Coccoidae (Hemiptera) (Viggiani 1987)

*Encarsia diaspidicola* (Silvestri, 1909)

Recorded from: Quilici et al. 2003

Host information: *Pseudaulacaspis pentagona* (Hemiptera, Diaspididae) (Liebregts et al. 1989)

*Encarsia formosa* Gahan, 1924

Recorded from: Vayssières et al. 2001

Host information: About 20 species in 8 genera of Aleyrodidae (Hemiptera) (Polaszek et al. 1992, Schauff et al. 1996)

*Encarsia lounsburyi* (Berlese & Paoli, 1916)

Recorded from: Quilici et al. 2003

Host information: *Parlatoria ziziphi*, *Aonidiella citrina* and *Parlatoria pergandii* (Hemiptera, Coccoidae) (Ghahari et al. 2004)

*Encarsia luteola* Howard, 1895

Recorded from: Vayssières et al. 2001

Host information: *Bemisia argentifolii*, *Bemisia tabaci* (Hemiptera, Aleyrodidae) (Gerling et al. 1987, Heinz and Parrella 1994)

*Encarsia nigricephala* Dozier, 1937

Recorded from: Vayssières et al. 2001

Host information: *Trialeurodes abulitonius*, *Trialeurodes floridensis*, *Trialeurodes vaporariorum* (Polaszek et al. 1999)

*Encarsia perniciosi* (Tower, 1913)

Recorded from: Quilici et al. 2003

Host information: *Quadraspidiotus perniciosus* (Baroffio 1997)

*Encarsia sophia* (Girault & Dodd, 1915)

Recorded from: Vayssières et al. 2001

Host information: *Bemisia tabaci*, *Trialeurodes vaporarium* (Hemiptera, Aleyrodidae) (Vayssières et al. 2001, Luo and Liu 2011)

*Encarsia tabacivora* Viggiani, 1985

Recorded from: Vayssières et al. 2001

Host information: 14 species from 9 genera of Aleyrodidae (Hemiptera) (Polaszek et al. 1992, Schauff et al. 1996)

##### Subfamily: Eretmocerinae

**Genus: *Eretmocerus*** Haldeman, 1850

*Eretmocerus hayati* Zolnerowich & Rose, 1998

Recorded from: Vayssières et al. 2001

Host information: *Bemisia tabaci*, *Trialeurodes vaporarium* (Hemiptera, Aleyrodidae) (Vayssières et al. 2001)

*Eretmocerus mundus* Mercet, 1931

Recorded from: Vayssières et al. 2001

Host information: *Bemisia argentifolii* (Hemiptera, Aleyrodidae) (Walker et al. 1999), *Bemisia tabaci*, *Trialeurodes vaporarium* (Hemiptera, Aleyrodidae) (Vayssières et al. 2001)

#### Family: CHALCIDIDAE

##### Subfamily: Chalcidinae

**Genus: *Brachymeria*** Westwood, 1829

*Brachymeria nephantidis* Gahan, 1930

Recorded from: Ramage et al. 2011

Host information: *Nephantis serinopa* (Lepidoptera, Xyloricitidae) (Joy et al. 1978), *Corcyra cephalonica* (Lepidoptera, Pyralidae) (Kapadia 1999)

*Brachymeria podagrica* (Fabricius, 1787)

Recorded from: Ramage et al. 2011

Host information: Moths in several families and Calyptrate Diptera such as: *Chrysomya albiceps* (Diptera, Calliphoridae) (Marchiori et al. 2002a) and *Sarcodexia lambens* (Diptera, Sarcophagidae) (Marchiori et al. 2002b)

##### Subfamily: Dirhinae

**Genus: *Dirhinus*** Dalman, 1818

*Dirhinus galesusaeformis* (Risbec, 1957)

Recorded from: Risbec 1957

Host information: Unknown

*Dirhinus giffardii* Silvestri, 1913

Recorded from: Vayssières et al. 2001

Host information: Diptera, Tephritidae (Noyes 2013)

*Dirhinus himalayanus* Westwood, 1836

Recorded from: Ramage et al. 2011

Host information: *Sarcophaga bullata* (Diptera, Sarcophagidae), *Hydrotaea aenescens*, *Stomoxys calcitrans*, and *Musca dometica* (Diptera, Muscidae) (Geden et al. 2006, Srinivasan and Amalraj 2003). *Dirhinus hymalayanus* is also a parasitoid of some Tephritidae (Diptera) pupae (Grissell and Schauff 1990, Sivinsky et al. 1997)

##### Subfamily: Epitraninae

**Genus: *Epitranus*** Walker, 1834

*Epitranus erythrogaster* Cameron, 1888

Recorded from: Ramage et al. 2011

Host information: *Corcyra cephalonica* (Lepidoptera, Pyralidae) (Boucek 2007)

*Epitranus evanioides* (Westwood, 1835)

Recorded from: Ramage et al. 2011

Host information: Unknown

##### Subfamily: Haltichellinae

**Genus: *Antrocephalus*** Kirby, 1883

*Antrocephalus crassipes* Masi, 1940

Recorded from: Ramage et al. 2011

Host information: Unknown

*Antrocephalus dividens* (Walker, 1860)

Recorded from: Ramage et al. 2011

Host information: Unknown

**Genus: *Hockeria*** Walker, 1834

*Hockeria fulvipes* Masi, 1917

Recorded from: Ramage et al. 2011

Host information: Unknown

**Genus: *Proconura*** Dodd, 1915

*Proconura eublemmae* (Steffan, 1951)

Recorded from: Ramage et al. 2011

Host information: *Eublemma gayneri* (Lepidoptera, Noctuidae) (Gahukar 1981)

#### Family: ENCYRTIDAE

##### Subfamily: Encyrtinae

**Genus: *Ageniaspis*** Dahlbom, 1857

*Ageniaspis citricola* Logvinovskaya, 1983

Recorded from: Quilici et al. 2003

Host information: *Phyllocnistis citrella* (Lepidoptera, Gracillariidae) (Edward and Hoy 1998, Hoy and Jessey 2004)

*Ageniaspis fuscicallis* (Dalman, 1920)

Recorded from: Quilici et al. 2003

Host information: *Yponomeuta malinellus* (Lepidoptera, Yponomeutidae) (Kuhlmann et al. 1998), *Acrolepia assectella* (Lepidoptera, Acrolepiidae) (Pralavorio et al. 1977)

**Genus: *Aloencyrtus*** Prinsloo, 1978

*Aloencyrtus obscuratus* (Waterston, 1917)

Recorded from: Quilici et al. 2003

Host information: Unknown. But *Aloencyrtus* appear to be exclusively parasitic in Coccidae (Hemiptera) (Prinsloo 2010)

**Genus: *Arrhenophagus*** Aurivillius, 1888

*Arrhenophagus chionaspidis* Aurivillius, 1888

Recorded from: Quilici et al. 2003

Host information: *Pseudaulacaspis pentagona* (Hemiptera, Diaspididae) (Kreiter et al. 2000, Goebel 1987)

**Genus: *Cheiloneurus*** Westwood, 1833

*Cheiloneurus cyanonotus* Waterston, 1917

Recorded from: Risbec 1957

Host information: *Phenacoccus manihoti* (Hemiptera, Pseudococcidae) (Fabres and Matile-Ferrero 1986)

**Genus: *Comperiella*** Howard, 1906

*Comperiella bifasciata* Howard, 1906

Recorded from: Quilici et al. 2003

Host information: *Aonidiella citrina*, *Aonidiella aurantii*, *Aspidiotus destructor* (Hemiptera, Diaspididae) (Flanders 1944, Tandon and Srivastava 1980)

**Genus: *Copidosoma*** Ratzeburg, 1844

*Copidosoma koehleri* Blanchard, 1940

Recorded from: Vayssières et al. 2001

Host information: *Phtorimea operculella* (Lepidoptera, Gelechiidae) (Dalaya and Patil 1973)

**Genus: *Diaphorencyrtus*** Hayat, 1981

*Diaphorencyrtus aligarhensis* (Shafee, Alam & Agarwal, 1975)

Recorded from: Quilici et al. 2003

Host information: *Diaphorina citri* (Hemiptera, Psyllidae) (Prinsloo 1985, Hoy and Nguyen 1998)

**Genus: *Habrolepis*** Förster, 1856

*Habrolepis rouxi* Compere, 1936

Recorded from: Quilici et al. 2003

Host information: *Anonidiella aurantii* (Hemiptera, Diaspididae) (Flanders 1942)

**Genus: *Homalotylus*** Mayr, 1876

*Homalotylus eytelweinii* (Ratzeburg, 1844)

Recorded from: Delpoux et al. 2013

Host information: Coccinellidae (Coleoptera) from the tribes Coccinellini, Chilocorini, and Psylloborini (Kuznetsov 1975, 1987, Trjapitzin 2011)

**Genus: *Metaphycus*** Mercet, 1917

*Metaphycus galbus* Annecke, 1964

Recorded from: Quilici et al. 2003

Host information: *Protopulvinaria pyriformis* (Hemiptera, Coccidae) (Robertson and De Villiers 1986)

**Genus: *Microterys*** Thomson, 1876

*Microterys nietneri* (Motschulsky, 1859)

Recorded from: Quilici et al. 2003

Host information: Hemiptera, Coccidae especially on citrus (Triapitsyn et al. 2008)

**Genus: *Psyllaephagus*** Ashmead, 1900

*Psyllaephagus pulvinatus* (Waterston, 1922)

Recorded from: Aubert 1975

Host information: *Trioza eryteae* (Homoptera, Psyllidae) (MacDaniel and Moran 1972)

**Genus: *Syrphophagus*** Ashmead, 1900

*Syrphophagus africanus* (Gahan, 1932)

Recorded from: Vayssières et al. 2001

Host information: Hyperparasitoid of some aphid parasitoids (e.g. *Lysiphlebus testaceipes*, *Phelinus ficusae*) (Saethre et al. 2011)

##### Subfamily: Tetracneminae

**Genus: *Coccidoxenoides*** Girault, 1915

*Coccidoxenoides perminutus* Girault, 1915

Recorded from: Quilici et al. 2003

Host information: *Planococcus ficus* (Hemiptera, Pseudococcidae) (Walton and Pringle 2005)

#### Family: EULOPHIDAE

##### Subfamily: Entedoninae

**Genus: *Chrysocharis*** Förster, 1856

*Chrysocharis bedius* (Walker, 1842)

Recorded from: Vayssières et al. 2001

Host information: *Liriomyza* species (Diptera, Agromyzidae) (Vayssières et al. 2001)

*Chrysocharis* sp.

Recorded from: Vayssières et al. 2001

Host information: *Liriomyza* species (Diptera, Agromyzidae) (Vayssières et al. 2001)

**Genus: *Chrysonotomyia*** Ashmead, 1904

*Chrysonotomyia pulcherrima* (Kerrich, 1970)

Recorded from: Quilici et al. 2003

Host information: *Procontarinia matteiana* (Diptera, Cecidomyiidae) (Sankaran and Mjeni 1985)

**Genus: *Neochrysocharis*** Kurdjumov, 1912

*Neochrysocharis formosa* (Westwood, 1833)

Recorded from: Vayssières et al. 2001

Host information: *Liriomyza* species (Diptera, Agromyzidae) (Vayssières et al. 2001)

*Neochrysocharis* sp.

Recorded from: Vayssières et al. 2001

Host information: *Liriomyza* species (Diptera, Agromyzidae) (Vayssières et al. 2001)

**Genus: *Platocharis*** Kerrich, 1969

*Platocharis coffeae* (Ferrière, 1936)

Recorded from: Kerrich 1969

Host information: Coffee leaf-miners (*Leucoptera* spp.) (Kerrich 1969)

##### Subfamily: Eulophinae

**Genus: *Cirrospilus*** Westwood, 1832

*Cirrospilus cinctiventris* Ferrière, 1936

Recorded from: Quilici et al. 2003

Host information: Leaf-miners from genus *Leucoptera* (Lepidoptera, Lyonetiidae) (Ferrière 1936)

*Cirrospilus crowei* Kerrich, 1969

Recorded from: Quilici et al. 2003

Host information: *Phyllocnistis citrella* (Lepidoptera, Gracillariidae) (Schauff et al. 1998)

**Genus: *Elachertus*** Spinola, 1811

*Elachertus insularis* (Risbec, 1957).

Recorded from: Risbec 1957

Host information: Unknown

**Genus: *Elasmus*** Westwood, 1833

*Elasmus masii* Ferrière, 1929

Recorded from: Risbec 1957

Host information: Recorded as both, primary and secondary parasitoid of Lepidoptera (Noyes and Yu 2012)

**Genus: *Euplectrus*** Westwood, 1832

*Euplectrus bebourensis* Risbec, 1957

Recorded from: Risbec 1957

Host information: Unknown. But *Euplectrus* are parasitoid of Lepidoptera larvae (Schauff and Janzen 2001)

**Genus: *Hamonia*** Risbec, 1957

*Hamonia reunionensis* Risbec, 1957

Recorded from: Risbec 1957

Host information: Unknown

*Hamonia sexdentata* Risbec, 1957

Recorded from: Risbec 1957

Host information: Unknown

*Hamonia sylvatica* Risbec, 1957

Recorded from: Risbec 1957

Host information: Unknown

**Genus: *Hemiptarsenus*** Westwood, 1833

*Hemiptarsenus varicornis* Girault, 1913

Recorded from: Vayssières et al. 2001

Host information: *Liriomyza trifolii* and other *Liriomyza* species (Diptera, Agromyzidae) (Arakaki and Kinjo 1998, Vayssières et al. 2001)

**Genus: *Notanisomorphella*** Girault, 1913

*Notanisomorphella borborica* (Giard, 1903)

Recorded from: Quilici et al. 2003

Host information: Leaf-miners from genus *Leucoptera* (Lepidoptera, Lyonetiidae) (Ferrière 1936)

**Genus: *Stenomesius*** Westwood, 1833

*Stenomesius belouvi* (Risbec, 1957)

Recorded from: Risbec 1957

Host information: Unknown

*Stenomesius masii* (Risbec, 1957)

Recorded from: Risbec 1957

Host information: Unknown

##### Subfamily: Tetrastichinae

**Genus: *Aprostocetus*** Westwood, 1833

*Aprostocetus ceroplastae* (Girault, 1916)

Recorded from: Quilici et al. 2003

Host information: *Ceroplastes destructor* (Hemiptera, Coccidae) (Wakgari and Giliomee 2001)

*Aprostocetus toddaliae* (Risbec, 1958)

Recorded from: Quilici et al. 2003

Host information: *Ceroplastes rusci* (Hemiptera, Coccidae) (Talebi et al. 2011)

**Genus: *Gyrolasia*** Förster, 1856

*Gyrolasia hellburgi* (Risbec, 1957)

Recorded from: Risbec 1957

Host information: Unknown

**Genus: *Nesolynx*** Ashmead, 1905

*Nesolynx phaeosoma* (Waterston, 1915)

Recorded from: Quilici et al. 2003

Host information: Hyperparasitoid on *Nephopterix beharella* (Lepidoptera, Pyralidae) (Quilici et al. 2003)

**Genus: *Oomyzus*** Rondani, 1870

*Oomyzus sokolowskii* (Kurdjumov, 1912)

Recorded from: Vayssières et al. 2001

Host information: *Plutella xylostella* (Lepidoptera, Yponomeutoidea) (Vayssières et al. 2001)

**Genus: *Quadrastichus*** Girault, 1913

*Quadrastichus erythrinae* Kim, 2004

Recorded from: Kim et al. 2004

Host information: Gall-inducer on *Erythrina* (Kim et al. 2004)

**Genus: *Tamaraxia*** Mercet, 1924

*Tamaraxia dryi* (Waterston, 1922)

Recorded from: Etienne and Aubert 1980

Host information: Ectoparasitoid of Psyllid larvae (Hemiptera, Psyllidae) (Quilici et al. 2003)

*Tamaraxia radiata* (Waterston, 1922)

Recorded from: Aubert et al. 1979

Host information: Ectoparasitoid of Psyllid larvae (Hemiptera, Psyllidae) (Quilici et al. 2003)

**Genus: *Tetrastichus*** Haliday, 1844

*Tetrastichus giffardianus* Silvestri, 1915

Recorded from: Vayssières et al. 2001

Host information: Endoparasitoids of several Tephritid larvae (Diptera) (Clausen et al. 1965)

*Tetrastichus gyrolasiaeformis* Risbec, 1957

Recorded from: Risbec 1957

Host information: Unknown

*Tetrastichus sesamiae* Risbec, 1951

Recorded from: Risbec 1957

Host information: Parasitoid of Lepidoptera (Noyes and Yu 2012)

#### Familiy: EUPELMIDAE

##### Subfamily: Eupelminae

**Genus: *Eupelmus*** Dalman, 1820

*Eupelmus* sp.

Recorded from: Vayssières et al. 2001

Host information: Unknown. But *Eupelmus* can be extremely polyphagous (Gibson 2011)

##### Familiy: EURYTOMIDAE

**Subfamily: Eurytominae**

**Genus: *Eurytoma*** Illiger, 1807

*Eurytoma ivohibei* Risbec, 1957

Recorded from: Risbec 1957

Host information: Unknown

*Eurytoma reunionensis* Risbec, 1957

Recorded from: Risbec 1957

Host information: Unknown

##### Familiy: ORMYRIDAE

**Genus: *Ormyrus*** Westwood, 1832

*Ormyrus australis* Risbec, 1957

Recorded from: Risbec 1957

Host information: Unknown

#### Family: PTEROMALIDAE

##### Subfamily: Eunotinae

**Genus: *Mesopeltita*** Ghesquière, 1946

*Mesopeltita truncatipennis* (Waterston, 1917)

Recorded from: Quilici et al. 2003

Host information: *Ceroplastes* sp., *Saissetia oleae*, *Saissetia somereni* and *Saissetia* sp. (Hemiptera, Coccidae) (Myartseva et al. 2004)

**Genus: *Moranila*** Cameron, 1883

*Moranila californica* (Howard, 1881)

Recorded from: Quilici et al. 2003

Host information: *Saissetia oleae*, *Ceroplastes floridensis* (Hemiptera: Coccidae) (Flanders 1958, Argov et al. 1992)

##### Subfamily: Miscogasterinae

**Genus: *Halticoptera*** Spinola, 1811

*Halticoptera* sp.

Recorded from: Vayssières et al. 2001

Host information: *Melanagromyza phaseoli*, *Liriomyza* spp. (Diptera, Agromyzidae) (Abul-Nasr and Assem 1968, Vayssières et al. 2001)

##### Subfamily: Pteromalinae

**Genus: *Muscidifurax*** Girault & Sanders, 1910

*Muscidifurax raptor* Girault & Sanders, 1910

Recorded from: Quilici et al. 2003

Host information: *Musca domestica*, *Stomoxys calcitrans* and other Synanthropic filth-breeding Diptera (Diptera, Muscidae) (Legner 1967, Zchori-Fein et al. 2000)

*Muscidifurax uniraptor* Kogan & Legner, 1970

Recorded from: Quilici et al. 2003

Host information: *Musca domestica*, *Stomoxys calcitrans* and other Synanthropic filth-breeding Diptera (Diptera, Muscidae) (Legner 1967, Zchori-Fein et al. 2000)

**Genus: *Pachyneuron*** Walker, 1833

*Pachyneuron* sp.

Recorded from: Vayssières et al. 2001

Host information: Unknown. But *Pachyneuron* are mostly Hyperparasitoid of Hemiptera, Aphididae, and some Diptera (Gibson 2001)

*Pachyneuron longiradius* Silvestri, 1915

Recorded from: Delvare G. unpublished determination (2013)

Host information: The identified *Dendrocerus* emerged from a specimen of *Rodolia chermesina* (Coleoptera, Coccinellidae) (Delvare 2013, unpublished data)

**Genus: *Ploskana*** Boucek, 1976

*Ploskana tenuis* Boucek, 1976

Recorded from: Vayssières et al. 2001

Host information: *Bactrocera cucurbitae* (Diptera, Tephritidae) (Vayssières et al. 2001)

**Genus: *Pteromalus*** Swederus, 1795

*Pteromalus* sp.

Recorded from: Vayssières et al. 2001

Host information: Unknown. But all species of *Pteromalus* are parasitoids of larvae and pupae of various holometabolous insects, for instance Lepidoptera, Coleoptera, gall forming Hymenoptera (Cynipidae, Tenthredinidae) and Diptera (Tephritidae) (Baur 2015)

**Genus: *Spodophagus*** Delvare & Rasplus, 1994

*Spodophagus lepidopterae* (Risbec, 1952)

Recorded from: Risbec 1952

Host information: *Spodoptera littoralis* (Lepidoptera, Noctuidae) (Risbec 1952, Delvare and Rasplus 1994)

**Genus: *Trichomalopsis*** Crawford, 1913

*Trichomalopsis* sp.

Recorded from: Vayssières et al. 2001

Host information: Diptera, Muscidae (Gibson and Floate 2001)

##### Subfamily: Spalangiinae

**Genus: *Spalangia*** Latreille, 1805

*Spalangia endius* Walker, 1839

Recorded from: Vayssières et al. 2001

Host information: *Musca domestica* (Diptera, Muscidae) (Ables and Shepard 1974), *Dacus ciliates* (Diptera, Tephritidae) (Vayssières et al. 2001)

*Spalangia gemina* Boucek, 1963

Recorded from: Vayssières et al. 2001

Host information: Diptera, Muscidae (Morgan et al. 1991), Tephritidae (Vayssières et al. 2001)

*Spalangia seyrigi* Risbec, 1952

Recorded from: Vayssières et al. 2001

Host information: *Dacus ciliatus* (Diptera, Tephritidae) (Vayssières et al. 2001)

##### Subfamily: Sycoecinae

**Genus: *Seres*** Waterston, 1919

*Seres bouceki* (Wiebes, 1981)

Recorded from: Wiebes 1981

Host information: *Ficus reflexa reflexa* Thunb. and *Ficus reflexa aldabrensis* (Baker) C.C. Berg (Wiebes 1981)

##### Subfamily: Sycoryctinae

**Genus: *Apocrypta*** Coquerel, 1855

*Apocrypta perplexa* Coquerel, 1855

Recorded from: Coquerel 1855

Host information: *Ficus mauritiana* Lam. (Moraceae) (Coquerel 1855)

**Genus: *Sycoryctes*** Mayr, 1885

*Sycoryctes anceps* Wiebes, 1981

Recorded from: Wiebes 1981

Host information: *Ficus densifolia* Miq. (Moraceae) (Wiebes 1981)

*Sycoryctes caelebs* Wiebes, 1975

Recorded from: Wiebes 1975

Host information: *Ficus densifolia* Miq. and *Ficus rubra* (Moraceae) (Wiebes 1981)

*Sycoryctes comparabilis* Wiebes, 1981

Recorded from: Wiebes 1981

Host information: *Ficus densifolia* Miq. (Moraceae) (Wiebes 1981)

*Sycoryctes remus* Wiebes, 1981

Recorded from: Bouček et al. 1981

Host information: *Ficus reflexa reflexa* Thunb. and *Ficus burkei* (Miq.) Miq. (Moraceae) (Bouček et al. 1981)

**Genus: *Sycoscapter*** Saunders, 1883

*Sycoscapter gibbus* Saunders, 1883

Recorded from: Saunders 1883

Host information: *Ficus politoria* Lam.and *Ficus lateriflora* Vahl (Moraceae) (Saunders 1883)

*Sycoscapter tibialis* Wiebes, 1981

Recorded from: Wiebes 1981

Host information: *Ficus rubra* Vahl (Moraceae) (Wiebes 1981)

**Genus: *Philotrypesis*** Förster, 1878

*Philotrypesis cnephaea* Wiebes, 1981

Recorded from: Wiebes 1981

Host information: *Ficus reflexa reflexa* Thunb. (Moraceae) (Wiebes 1981)

**Genus: *Watshamiella*** Wiebes, 1981

*Watshamiella fictitia* Wiebes, 1981

Recorded from: Wiebes 1981

Host information: *Ficus rubra* Vahl (Moraceae) (Wiebes 1981)

*Watshamiella lucens* Wiebes, 1981

Recorded from: Wiebes 1981

Host information: *Ficus densifolia* Miq. (Moraceae) (Wiebes 1981)

#### Family: SIGNIPHORIDAE

**Genus: *Chartocerus*** Motschulsky, 1859

*Chartocerus* sp.

Recorded from: Delvare G. unpublished determination (2013)

Host information: In 2013 *Dendrocerus wollastoni* emerged from a specimen of *Rodolia chermesina* (Coleoptera, Coccinellidae) in Reunion Island (Delvare 2013, unpublished data)

#### Family: TORYMIDAE

##### Subfamily: Megastigminae

**Genus: *Megastigmus*** Dalman, 1820

*Megastigmus transvaalensis* (Hussey, 1956)

Recorded from: Habeck et al. 1989

Host information: Develops on plants in the genera *Rhus* and *Schinus* (Anacardiaceae) (Hussey 1956, Habeck et al. 1989)

#### Family: TRICHOGRAMMATIDAE

##### Subfamily: Trichogrammatinae

**Genus: *Trichogramma*** Westwood, 1833

*Trichogramma chilonis* Ishii, 1941

Recorded from: Vayssières et al. 2001

Host information: *Plutella xylostella* (Lepidoptera, Plutellidae) (Vayssières et al. 2001)

### Superfamily: CYNIPOIDEA

#### Family: FIGITIDAE

##### Subfamily: Charipinae

**Genus: *Phaenoglyphis*** Förster, 1869

*Phaenoglyphis villosa* (Hartig, 1841)

Recorded from: Evenhuis and Barbotin 1977

Host information: *Aphis nerii* (Hemiptera, Aphididae) (Evenhuis and Barbotin 1977), Hyperparasitoid of Aphididae (Hemiptera) via Aphidiinae (Hymenoptera, Braconidae) (Pujade-villar et al. 2007)

##### Subfamily: Eucoilinae

**Genus: *Aganaspis*** Lin, 1987

*Aganaspis daci* (Weld, 1951)

Recorded from: Etienne 1975

Host information: *Bactrocera dorsalis* (Clausen et al. 1965), *Anastrepha suspensa* (Nuñez-Bueno 1982), *Ceratitis capitata* and *Ceratitis rosa* (Diptera, Tephritidae) (Etienne 1975)

**Genus: *Leptopilina*** Förster, 1869

*Leptopilina freyae* Allemand & Nordlander, 2003

Recorded from: Allemand et al. 2003

Host information: *Drosophila* ssp. (Diptera, Drosophilidae) (Allemand et al. 2003)

*Leptopilina guineanensis* Allemand & Nordlander, 2003

Recorded from: Allemand et al. 2003

Host information: *Drosophila* ssp. (Diptera, Drosophilidae) (Allemand et al. 2003)

*Leptopilina orientalis* Allemand & Nordlander, 2003

Recorded from: Allemand et al. 2003

Host information: *Drosophila* ssp. (Diptera, Drosophilidae) (Allemand et al. 2003)

*Leptopilina victoriae* Nordlander, 1980

Recorded from: Allemand et al. 2003

Host information: *Drosophila* ssp. (Diptera, Drosophilidae) (Allemand et al. 2003)

**Genus: *Rhoptromeris*** Förster, 1869

*Rhoptromeris bupalus* Quinlan, 1986

Recorded from: Quinlan 1986

Host information: Parasitoid of Chloropidae (Diptera) on grass and fungi (van Noort et al. 2015)

*Rhoptromeris crito* Quinlan, 1986

Recorded from: Quinlan 1986, Madl and van Achterberg 2014

Host information: Parasitoid of Chloropidae (Diptera) on grass and fungi (van Noort et al. 2015)

### Superfamily: CHRYSIDOIDEA

#### Family: BETHYLIDAE

##### Subfamily: Bethylinae

**Genus: *Goniozus*** Förster, 1856

*Goniozus indicus* (Ashmead, 1903)

Recorded from: Polaszek et al. 1994

Host information: *Maliarpha separatiella* (Lepidoptera, Pyralidae), *Cryptophlebia leucotreta*, *Cryptophlebia peltastica*, *Cryptophlebia semilunana*, *Eccopsis praecedens*, *Strepsicrates rhothia* (Lepidoptera, Olethreutidae) (Azevedo et al. 2010)

#### Family: CRHYSIDIDAE

##### Subfamily: Amiseginae

**Genus: *Senesega*** Kimsey, 2005

*Senesega attiei* Kimsey, 2005

Recorded from: Kimsey 2005

Host information: Probably parasitoid of Phasmatodea (Kimsey 2005)

##### Subfamily: Chrysidinae

**Genus: Chrysis** Linnaeus, 1761

*Chrysis gheudei* Guérin-Méneville, 1842

Recorded from: Azevedo et al. 2010. The record from Reunion is probably a misidentification of *Chrysis lincea* Fabricius 1775 (Azevedo et al. 2010)

Host information: *Delta reginum* (Hymenoptera, Vespidae, Eumeninae) and *Sceliphron fuscum* (Hymenoptera, Sphecidae) (Azevedo et al. 2010)

**Genus: *Praestochrysis*** Linsenmaier, 1959

*Praestochrysis lusca* (Fabricius, 1804)

Recorded from: Bordage 1912

Host information: *Chalybion madecassum*, *Sceliphron fuscum* (Hymenoptera, Sphecidae) (Azevedo et al. 2010)

#### Family: DRYINIDAE

##### Subfamily: Anteoninae

**Genus: *Anteon*** Jurine, 1807

*Anteon reunionense* Olmi, 1987

Recorded from: Olmi 1987

Host information: Unknown. But Dryinidae (including *Anteon*) are parasitoids of Homoptera auchenorrhyncha and commonly called “Cicada wasps”(Azevedo et al. 2010)

##### Subfamily: Gonatopodinae

**Genus: *Gonatopus*** Ljungh, 1810

*Gonatopus similis* Brues, 1906

Recorded from: Olmi 1987a

Host information: *Dicranotropis muiri*, *Perkinsiella saccharicida* (Hemiptera, Delphacidae) (Azevedo et al. 2010)

### Superfamily: PLATYGASTROIDEA

#### Family: PLATYGASTRIDAE

##### Subfamily: Platygastrinae

**Genus: *Leptacis*** Förster, 1856

*Leptacis risbeci* Masner, 1960

Recorded from: Masner 1960, Madl 2016

Host information: Parasitoids of Cecidomyiidae (Diptera) (Buhl 2011)

**Genus: *Synopeas*** Förster, 1856

*Synopeas pauliani* (Risbec, 1957)

Recorded from: Risbec 1957, Masner 1960

Host information: Parasitoids of Cecidomyiidae (Diptera) (Buhl 2011)

#### Family: SCELIONIDAE

##### Subfamily: Scelioninae

**Genus: Macroteleia** Westwood, 1935

*Macroteleia insularis* (Risbec, 1957)

Recorded from: Risbec 1957

Host information: Unknown

**Genus: *Styloteleia*** Kieffer, 1916

*Styloteleia gibbosa* Risbec, 1957

Recorded from: Risbec 1957

Host information: Unknown

##### Subfamily: Telenominae

**Genus: *Telenomus*** Haliday, 1833

*Telenomus busseolae* Gahan, 1922

Recorded from: Rao and Nagaraja 1969

Host information: Parasitizes eggs of *Busseola fusca*, *Sesamia calamistis* and *Sesamia cretica* (Lepidoptera, Pyralidae) (Samin and Asgari 2012)

### Superfamily: DIAPRIOIDEA

#### Family: DIAPRIIDAE

##### Subfamily: Diapriinae

**Genus: *Trichopria*** Ashmead, 1893

*Trichopria belouvi* (Risbec, 1957)

Recorded from: Risbec 1957, Notton 2004, Madl 2015

Host information: Unknown. But *Trichopria* are parasitoids of Tachinidae (Diptera) (Rajmohana 2006) and Tephritidae (Diptera) (Vayssières et al. 2001)

*Trichopria inconspicua* (Kieffer, 1905)

Recorded from: Huggert 1977, Madl 2015

Host information: Unknown. But *Trichopria* are parasitoids of Tachinidae (Diptera) (Rajmohana 2006) and Tephritidae (Diptera) (Vayssières et al. 2001)

*Trichopria jeanneli* Notton, 2004

Recorded from: Risbec 1957, Madl 2015

Host information: Unknown. But *Trichopria* are parasitoids of Tachinidae (Diptera) (Rajmohana 2006) and Tephritidae (Diptera) (Vayssières et al. 2001)

*Trichopria scotti* (Kieffer, 1912)

Recorded from: Risbec 1957

Host information: Unknown. But *Trichopria* are parasitoids of Tachinidae (Diptera) (Rajmohana 2006) and Tephritidae (Diptera) (Vayssières et al. 2001)

*Trichopria variabilis* (Risbec, 1950)

Recorded from: Risbec 1957, Madl 2015

Host information: Unknown. But *Trichopria* are parasitoids of Tachinidae (Diptera) (Rajmohana 2006) and Tephritidae (Diptera) (Vayssières et al. 2001)

### Superfamily: CERAPHRONOIDEA

#### Family: CERAPHRONIDAE

**Genus: *Aphanogmus*** Thomson, 1858

*Aphanogmus fijiensis* (Ferrière, 1933)

Recorded from: Polaszek and LaSalle 1995, Madl 2015

Host information: *Cotesia sesamiae* (Hymenoptera, Braconidae) (Polaszek and LaSalle 1995)

**Genus: *Ceraphron*** Jurine, 1807

*Ceraphron amphimelas* Dessart, 1989

Recorded from: Dessart 1989, Madl 2015

Host information: Hemiptera, Diaspididae (Dessart 1989)

#### Family: MEGASPILIDAE

##### Subfamily: Megaspilinae

**Genus: *Dendrocerus*** Ratzeburg, 1852

*Dendrocerus wollastoni* (Dodd, 1920)

Recorded from: Dessart 1984, Madl 2015

Host information: Neuroptera, Chrysopidae (Dessart 1984). In 2013 *Dendrocerus wollastoni* emerged from a specimen of *Rodolia chermesina* (Coleoptera, Coccinellidae) in Reunion Island (Delvare 2013, unpublished data)

*Dendrocerus* sp.

Recorded from: Delvare G. unpublished determination (2013)

Host information: Cf. *Dendrocerus wollastoni*.

### Superfamily: EVANIOIDEA

#### Family: EVANIIDAE

**Genus: *Evania*** Fabricius, 1775

*Evania appendigaster* (Linnaeus, 1758)

Recorded from: Madl and Ganeshan 2008

Host information: Oviposits within the oothecae of *Periplaneta americana* and possibly that of *Periplaneta australasiae* and *Rhyparobia maderae* (Dictyoptera, Blattidae) (Madl and Ganeshan 2008)

**Genus: *Prosevania*** Kieffer, 1911

*Prosevania erythrosoma* (Schletterer, 1886)

Recorded from: Recorded from Rodrigues and Mauritious, but not yet from Reunion Island (Madl and Ganeshan 2008)

Host information: Oviposits within the oothecae of *Blatta orientalis* (Dictyoptera, Blattidae) (Madl and Ganeshan 2008)

**Table 1.** List of Ichneumonoidea recorded from Reunion Island.

Familly	Subfamilly	Genus / Species	General host information	Recorded from
**BRACONIDAE**	**Agathidinae**	*Biroia costata* (Brullé, 1846)	Unknown	Granger 1949
		*Camptothlipsis curticornis* Granger, 1949	*Nephopterix beharella*	Quilici et al. 2003
		*Coccygidium lutea* (Brullé, 1846)	*Condica conductaMythimna curvuvula Mythimna loreyi*	Brullé 1846
	**Alysiinae**	*Adelphenaldis grimmorum* Fischer, 2014	Unknown	Fischer 2014
		*Adelphenaldis nanocorpus* Fischer, 2014	Unknown	Fischer 2014
		*Aphaereta* sp.	Diptera Cyclorrhapha	Quilici et al. 2003
		*Asobara diadegmae* Fischer, 2014	*Diadegma* sp. (Ichneumonidae)	Fischer 2014
		*Cratospila sinenotaulis* Fischer, 2014	Unknown	Fischer 2014
		*Dacnusa sibirica* Telenga, 1934	Agromyzidae	Vayssières et al. 2001
		*Dacnusa* sp.	Agromyzidae	Vayssières et al. 2001
**BRACONIDAE**	**Alysiinae**	*Dinotrema candidiapex* Fischer, 2014	Unknown	Fischer 2014
		*Synaldis dugainensis* Fischer, 2014	Unknown	Fischer 2014
		*Synaldis robusticeps* Fischer, 2014	Unknown	Fischer 2014
	**Aphidiinae**	*Aphidius camerunensis* Mackauer, 1966	*Sitobion*	Stary et al. 1994
		*Aphidius colemani* Viereck, 1912	Aphididae	Vayssières et al. 2001
		*Aphidius rosae* Haliday, 1833	*Macrosiphum rosaeSitobion fragariae*	Stary et al. 1977
		*Aphidius seyrigi* Granger, 1949	Aphididae	Benoit 1957
		*Diaeretiella rapae* (McIntosh, 1855)	Aphididae	Quilici et al. 1988
		*Praon* sp.	*Aphis* sp.	Vayssières et al. 2001
	**Braconinae**	Bracon (Habrobracon) hebetor (Say, 1836)	Crambidae, Lycaenidae, Pyralidae (Lep.)	Quilici et al. 2003
		*Iphiaulax didymus* (Brullé, 1846)	Unknown	Brullé 1846
	**Charmontinae**	*Charmon ramagei* Rousse, 2013	Unknown	Rousse 2013
	**Cheloninae**	Chelonus (Microchelonus) curvimaculatus Cameron, 1906	*Pectinophora gossypiella*, *Trichoplusia ni*	Quilici et al. 2003 Braet et al. 2012
		Chelonus (Microchelonus) matilei Braet & Rousse, 2012	Unknown	Braet et al. 2012
		Chelonus (Chelonus) mayi Braet & Rousse, 2012	Unknown	Braet et al. 2012
		Chelonus (Microchelonus) merdicus Rousse & Braet, 2012	Unknown	Braet et al. 2012
		*Chelonus* sp.	Unknown	Quilici et al. 2003
	**Doryctinae**	*Ipodoryctes* sp.	Coleoptera	Zaldivar-Riveron et al. 2008
		*Rinamba opacicollis* Cameron, 1912	Coleoptera	Braet 2001
	**Euphorinae**	*Centistes caloupile* Rousse & Braet, 2012	Unknown	Rousse and Braet 2012
		*Chrysopophthorus* sp.	Chrysopidae	Semeria and Quilici 1986
		*Cosmophorus merdiculatus* Rousse & Braet, 2012	Scolytinae	Rousse and Braet 2012
		*Leiophron sarahae* Rousse & Braet, 2012	Miridae or Psocoptera	Rousse and Braet 2012
		*Leiophron yaeli* Rousse & Braet, 2012	Miridae or Psocoptera	Rousse and Braet 2012
		*Meteorus anthracnemis* de Saeger, 1946	Lepidoptera	Rousse and Braet 2012
		*Meteorus comonile* Rousse & Braet, 2012	Lepidoptera	Rousse and Braet 2012
		*Meteorus pulchricornis* Rousse & Braet, 2012	Lepidoptera	Rousse and Braet 2012
**BRACONIDAE**	**Euphorinae**	*Syntretus massale* Rousse & Braet, 2012	Hymenoptera	Rousse and Braet 2012
	**Gnamptodontinae**	*Gnamptodon legorskyi* Fischer, 2014	Nepticulidae	Fischer 2014
	**Microgastrinae**	*Apanteles aff. acutissimus* Granger, 1949	*Diaphana indica*	Vayssières et al. 2001
		*Apanteles bordagei* Giard, 1902	*Crobylophora daricellaLeucoptera caffeinaLeucoptera mayricki*	Rousse and Gupta 2013
		*Apanteles fontinalis* de Saeger, 1944	Lepidoptera	Rousse and Gupta 2013
		*Apanteles galleriae* Wilkinson, 1932	Lepidoptera, Pyralidae	Rousse and Gupta 2013
		*Apanteles minatchy* Rousse & Gupta, 2013	Lepidoptera	Quilici et al. 2003
		*Apanteles nigrofemoratus* Granger, 1949	Lepidoptera, Cheurotidae	Rousse and Gupta 2013
		*Apanteles phasmina* Rousse, 2013	Lepidoptera	Rousse and Gupta 2013
		*Apanteles romei* Rousse, 2013	*Diaphina* sp.	Rousse and Gupta 2013
		*Cotesia flavipes* Cameron, 1891	Lepidoptera	Kfir et al. 2002
		*Cotesia plutellae* (Kurdumov, 1912)	Lepidoptera	Rousse and Gupta 2013
		*Cotesia ruficrus* (Haliday, 1834)	Lepidoptera	Rousse and Gupta 2013
		*Cotesia sesamiae* (Cameron, 1906)	Lepidoptera, Noctuidae, Pyralidae	Van Achterberg and Polaszek 1996
		*Cotesia xavieri* Rousse, 2013	Lepidoptera	Rousse and Gupta 2013
		*Diolcogaster austrina* (Wilkinson, 1929)	Lepidoptera	Aubert 1966
		*Diolcogaster curticornis* (Granger, 1949)	Lepidoptera	Rousse and Gupta 2013
		*Distatrix belliger* (Wilkinson, 1929)	Lepidoptera	Rousse and Gupta 2013
		*Distatrix yunae* Rousse & Gupta, 2013	Lepidoptera	Rousse and Gupta 2013
		*Dodogaster grangeri* Rousse, 2013	Lepidoptera	Rousse and Gupta 2013
		*Dolichogenidea ashoka* Rousse, 2013	Lepidoptera	Rousse and Gupta 2013
		*Dolichogenidea broadi* Rousse, 2013	Lepidoptera	Rousse and Gupta 2013
		*Dolichogenidea lumba* Rousse & Gupta, 2013	Lepidoptera	Rousse and Gupta 2013
		*Dolichogenidea uru* Rousse & Gupta, 2013	Lepidoptera	Rousse and Gupta 2013
**BRACONIDAE**	**Microgastrinae**	*Dolichogenidea villemantae* Rousse, 2013	Lepidoptera	Rousse and Gupta 2013
		*Exoryza safranum* Rousse & Gupta, 2013	Lepidoptera	Rousse and Gupta 2013
		*Glyoptapanteles antsirabensis* (Granger, 1949)	Lepidoptera	Rousse and Gupta 2013
		*Glyptapanteles chidra* Rousse & Gupta, 2013	Lepidoptera	Rousse and Gupta 2013
		*Glyptapanteles ficus* (Granger, 1949)	*Naroma signifera*	Rousse and Gupta 2013
		*Glyptapanteles subandinus* (Blanchard, 1947)	*Achyra bifidalisPhthorimaea opercullelaScrobipalpula absoluta*	Rousse and Gupta 2013
		*Microplitis subsulcatus* Granger, 1949	Lepidoptera	Rousse and Gupta 2013
		*Nyereria ganges* Rousse & Gupta, 2013	Lepidoptera	Rousse and Gupta 2013
		*Nyereria mayurus* Rousse & Gupta, 2013	Lepidoptera	Rousse and Gupta 2013
		*Parapanteles covino* Rousse & Gupta, 2013	Lepidoptera	Rousse and Gupta 2013
		*Parapanteles darignac* Rousse & Gupta, 2013	Lepidoptera	Rousse and Gupta 2013
		*Venanides curticornis* (Granger, 1949)	Lepidoptera	Rousse and Gupta 2013
		*Wilkinsonellus narangahus* Rousse & Gupta, 2013	Unknown	Rousse and Gupta 2013
	**Opiinae**	*Diachasmimorpha fullawayi* (Silvestri, 1913)	*Ceratitis capitata*	Quilici et al. 2003
		*Diachasmimorpha tryoni* (Cameron, 1911)	Diptera, Tephritidae	Hurtrel et al. 1999
		*Fopius arisanus* (Sonan, 1932)	Diptera, Tephritidae	Quilici et al. 2003
		*Opius dissitus* Muesebeck, 1963	Diptera, Agromyzidae	Vayssières et al. 2001
		Opius (Utetes) coriacitergum Fischer, 2014	Diptera Cyclorrhapha	Fischer 2014
		Opius (Tolbia) karlmayi Fischer, 2014	Diptera Cyclorrhapha	Fischer 2014
		Opius (Gastrosema) laticrenis Fischer, 2014	Diptera Cyclorrhapha	Fischer 2014
		Opius (Opius) raoiformis Fischer, 2014	Diptera Cyclorrhapha	Fischer 2014
		Opius (Utetes) semipilosus Fischer, 2014	Diptera Cyclorrhapha	Fischer 2014
		Opius (Gastrosema) waterloti Granger, 1949	Diptera Cyclorrhapha	Fischer and Madl 2008
		*Psyttalia cosyrae* [1] (Wilkinson, 1927)	*Dacus* sp.	–
**BRACONIDAE**	**Opiinae**	*Psyttalia distinguenda* (Granger, 1949)	Diptera Cyclorrhapha	Wharton et al. 1999
		*Psyttalia fletcheri* (Sylvestri, 1916)	Diptera Cyclorrhapha	Hurtrel et al. 1999
		*Psyttalia insignipennis* (Granger, 1949)	*Ceratitis capitataCeratitis catoiriiNeoceratitis cyanescens*	Wharton et al. 1999
		*Psyttalia phaeostigma* (Wilkinson, 1927)	*Dacus ciliatusDacus demmerezi*	Fischer 1987
		*Psyttalia sanctamarina* (Fischer, 1980)	*Spathulina aroleuca*	Fischer 1980
		*Psyttalia subsulcata* (Granger, 1949)	*Spathulina aroleuca*	Fischer and Madl 2008
**ICHNEUMONIDAE**	**Banchinae**	*Himertosoma bebourensis* (Benoit, 1957)	Lepidoptera, Tortricidae, Gelechiidae	Benoit 1957
		*Himertosoma striata* (Seyrig, 1932)	Lepidoptera, Tortricidae, Gelechiidae	Rousse and Villemant 2012
	**Campopleginae**	*Campoplex techer* Rousse & Villemant, 2012	Lepidoptera, Coleoptera or Hymenoptera	Rousse and Villemant 2012
		*Diadegma insulare* (Cresson, 1865)	Lepidoptera, Plutellidae, Pyralidae	Rousse and Villemant 2012
		*Diadegma mollipla* (Holmgren, 1868)	Lepidoptera, Plutellidae, Gelechiidae	Vayssières et al. 2001
		*Dusona douraguia* Rousse & Villemant, 2012	Symphyta or Lepidoptera	Rousse and Villemant 2012
		*Dusona pauliani* (Benoit, 1957)	Symphyta or Lepidoptera	Rousse and Villemant 2012
		*Enytus huet* Rousse & Villemant, 2012	Lepidoptera, Coleoptera or Hymenoptera	Rousse and Villemant 2012
		*Eriborus cadjee* Rousse & Villemant, 2012	Unknown.	Rousse and Villemant 2012
		*Eriborus pallipes* (Brullé, 1846)	Unknown.	Rousse and Villemant 2012
		*Hyposoter reunionis* (Benoit, 1957)	Lepidoptera, Coleoptera or Hymenoptera	Benoit 1957
		*Venturia canescens* (Gravenhorst, 1829)	Lepidoptera	Rousse and Villemant 2012
		*Xanthocampoplex huberti* Rousse & Villemant, 2012	Lepidoptera, Gracillariidae, Pyralidae	Rousse and Villemant 2012
	**Cremastinae**	*Pristomerus rivier* Rousse & Villemant, 2012	*Plutella xylostella*, *Prophantis smaragdina*	Rousse and Villemant 2012
		*Temelucha basiornata* (Cameron, 1911)	Diptera, Diopsidae	Rousse and Villemant 2012
		*Temelucha labusi* Rousse & Villemant, 2012	Lepidoptera	Rousse and Villemant 2012
	**Cremastinae**	*Temelucha minuta* (Morley, 1912)	Lepidoptera	Rousse and Villemant 2012
**ICHNEUMONIDAE**	**Cremastinae**	*Temelucha pestifer* (Morley, 1913)	Lepidoptera	Benoit 1957
		*Temelucha talibarti* Rousse, Villemant & Seyrig, 2011	Lepidoptera	Rousse and Villemant 2012
		*Temelucha* sp. 1	Lepidoptera	Rousse and Villemant 2012
		*Temelucha* sp. 2	Lepidoptera	Rousse and Villemant 2012
		*Trathala annulicornis* (Tosquinet, 1896)	*Leucinodes orbonalis*	Rousse and Villemant 2012
		*Trathala flavoorbitalis* (Cameron, 1907)	*Leucinodes orbonalis*	Rousse and Villemant 2012
	**Cryptinae**	*Acrolyta dindar* Rousse & Villemant, 2012	Unknown.	Rousse and Villemant 2012
		*Diatora albiscapus* (Seyrig, 1952)	*Apanteles*	Rousse and Villemant 2012
		*Paraphylax mussar* Rousse & Villemant, 2012	*Heliothis armigera*	Rousse and Villemant 2012
		*Paraphylax transversatoria* (Seyrig, 1952)	*Heliothis armigera*	Rousse and Villemant 2012
		*Phygadeuon nativel* Rousse & Villemant, 2012	*Musca domesticaRhagoletis cerasi*	Rousse and Villemant 2012
	**Diplazontinae**	*Diplazon laetatorius* (Fabricius, 1781)	Diptera, Syrphidae	Saussure 1892
	**Ichneumoninae**	*Crytea albitrochanterata* (Heinrich, 1938)	*Aletia pallens Despressaria heracliana*	Rousse and Villemant 2012
		*Ichneumon unicinctus* Brullé, 1846	Lepidoptera, Noctuidae, Amphipyrinae	Rousse and Villemant 2012
	**Mesochorinae**	*Mesochorus cariniferus* Benoit, 1955	*Phryganidia california*	Rousse and Villemant 2012
		*Mesochorus* sp.	*Phryganidia california*	Rousse and Villemant 2012
	**Metopiinae**	*Triclistus aitkeni* (Cameron, 1897)	Unknown.	Rousse and Villemant 2012
	**Ophioninae**	*Enicospilus angustatus* (Brullé, 1846)	Lepidoptera	Bordage 1914
		*Enicospilus betanimenus* (de Saussure, 1892)	Lepidoptera, Noctuidae	Rousse and Villemant 2012
		*Enicospilus dolosus* (Tosquinet, 1896)	Lepidoptera, Noctuidae, Crambidae	Rousse and Villemant 2012
		*Enicospilus dubius* (Tosquinet, 1896)	Lepidoptera, Noctuidae, Plusiidae	Rousse and Villemant 2012
		*Eniscopilus grandiflavus* Townes, 1973	Lepidoptera	Rousse and Villemant 2012
		*Enicospilus helvolus* Gauld & Mitchell, 1978	*Anomis leona*	Rousse and Villemant 2012
		*Enicospilus hova* Gauld & Mitchell, 1978	Lepidoptera	Gauld and Mitchell 1978
**ICHNEUMONIDAE**	**Ophioninae**	*Enicospilus mauritii* (de Saussure, 1892)	Lepidoptera	Bordage 1914
		*Enicospilus rufus* (Brullé, 1846)	*Ctenoplusia limbirenaTrigonodes hyppasia*	Benoit 1957
		*Enicospilus ruscus* Gauld & Mitchell, 1978	Lepidoptera, Noctuidae	Rousse and Villemant 2012
		*Enicospilus sesamiae* Delobel, 1974	Lepidoptera, Brachodidae, Noctuidae	Zwart 1998
		*Enicospilus transvaalensis* Cameron, 1911	*Mythimna* sp. *Sesamia calamistis*	Rousse and Villemant 2012
		*Enicospilus vitry* Rousse & Villemant, 2012	Lepidoptera	Rousse and Villemant 2012
	**Orthocentrinae**	*Megastylus vagabundus* Seyrig, 1934	Diptera or Hymenoptera	Rousse and Villemant 2012
		*Orthocentrus urbanus* Seyrig, 1934	Diptera, Coleoptera or Hymenoptera	Rousse and Villemant 2012
		*Pantisarthrus isolatus* (Benoit, 1957)	Unknown.	Benoit 1957
		*Proclitus ligatus* (Seyrig, 1934)	Diptera or Lepidoptera	Rousse and Villemant 2012
		*Stenomacrus payet* Rousse & Villemant, 2012	Unknown.	Rousse and Villemant 2012
		*Tariqia stellaris* Rousse & Villemant, 2012	Unknown.	Rousse and Villemant 2012
	**Pimplinae**	*Echthromorpha spinator* (Fabricius, 1798)	*Nagia linteola*	Rousse and Villemant 2012
		*Itoplectis albipes* Seyrig, 1932	Unknown.	Rousse and Villemant 2012
		*Xanthophenax defector* Seyrig, 1932	*Hypsipyla scabruscelella*	Rousse and Villemant 2012
		*Xanthophenax pacificator* Seyrig, 1932	*Hypsipyla scabruscelella*	Rousse and Villemant 2012
		*Xanthophenax xanthomelas* (Brullé, 1846)	*Hypsipyla scabruscelella*	Rousse and Villemant 2012
		*Xanthopimpla stemmator* (Thunberg, 1824)	Lepidoptera, Pyralidae	Kfir et al. 2002
		*Zatypota inexpectata* Seyrig, 1932	Araneae or Lepidoptera	Rousse and Villemant 2012
	**Tersilochinae**	*Aneuclis larga* Khalaim, 2009	Unknown.	Rousse and Villemant 2012
		*Diaparsis ramassamy* Rousse & Villemant, 2012	Coleoptera, Symphyta	Rousse and Villemant 2012
	**Tryphoninae**	*Netelia melanopus* (Brullé, 1846)	Lepidoptera, Tortricidae, Pyralidae, Noctuidae	Saussure 1892
		*Netelia* sp.	Lepidoptera	Rousse and Villemant 2012

[1] Fischer (1987) recorded it from Reunion, because «Réunion, Mayotte» is indicated on the label

**Table 2.** List of the Chalcidoidea recorded from Reunion Island.

Familly	Subfamilly	Genus / Species	General host information	Recorded from
**AGAONIDAE**	**Agaoninae**	*Elisabethiella reflexa* Wiebes, 1975	*Ficus reflexa* and *Ficus aldabrensis* (Moraceae)	Wiebes 1975
		*Nigeriella avicola* Wiebes, 1975	*Ficus rubra* (Moraceae)	Wiebes 1975
		*Platyscapa etiennei* Wiebes, 1977	*Ficus salicifolia* (Moraceae)	Wiebes 1977
	**Kradibiinae**	*Ceratosolen coecus* (Coquerel, 1855)	*Ficus mauritiana* (Moraceae)	Coquerel 1855
		*Kradibia etiennei* Wiebes, 1990	*Ficus lateriflora* (Moraceae)	Wiebes and Gompton 1990
	**Sycophaginae**	*Sycophaga explorator* (Coquerel, 1855)	Uknown Moraceae	Coquerel 1855
**APHELINIDAE**	**Aphelininae**	*Aphelinus asychis* (Walker, 1839)	Aphididae (Hemiptera)	Vayssières et al. 2001
		*Aphelinus gossypii* Timberlake, 1924	Aphididae (Hemiptera)	Quilici et al. 2003
		*Aphelinus mariscusae* (Risbec, 1957)	Aphididae (Hemiptera)	Risbec 1957
		*Aphelinus varipes* (Förster, 1841)	Aphididae (Hemiptera)	Vayssières et al. 2001
		*Aphytis africanus* (Quednau, 1964)	*Aonidiella aurantii* (Hemiptera: Diaspididae)	Quilici et al. 2003
		*Aphytis coheni* (DeBach, 1960)	*Aspidiotus nerii* (Hemiptera: Diaspididae)	Quilici et al. 2003
		*Aphytis holoxanthus* (DeBach, 1960)	Diaspididae (Hemiptera)	Quilici et al. 2003
		*Aphytis lepidosaphes* (Compere, 1955)	*Lepidosaphes beckii* (Hemiptera: Diaspididae)	Quilici et al. 2003
		*Aphytis lignanensis* (Compere, 1955)	*Aonidiella aurantii*, *Pseudaulacaspis pentagona* (Hemiptera: Diaspididae)	Quilici et al. 2003
		*Aphytis melinus* (DeBach, 1959)	*Aonidiella aurantii* (Hemiptera: Diaspididae)	Quilici et al. 2003
		*Marietta leopardina* (Motschulsky, 1863)	Hymenoptera	Quilici et al. 2003
	**Calesinae**	*Cales noacki* Howard, 1907	*Aleurotrachelus atratus*, *Aleurothrixus floccosus* (Hemiptera: Aleyrodidae)	Etienne 1977
	**Coccophaginae**	*Coccophagus ceroplastae* (Howard, 1985)	Coccoidae, Diaspididae or Pseudococcidae (Hemiptera)	Quilici et al. 2003
		*Coccophagus cowperi* (Girault, 1917)	*Saissetia* (Hemiptera: Coccidae)	Quilici et al. 2003
		*Coccophagus rusti* (Compere, 1928)	*Saissetia oleae* (Hemiptera: Coccidae)	Quilici et al. 2003
		*Encarsia acaudaleyrodis* Hayat, 1976	Aleyrodidae (Hemiptera)	Vayssières et al. 2001
		*Encarsia brasiliensis* (Hempel, 1904)	Aleyrodidae (Hemiptera)	Vayssières et al. 2001
		*Encarsia citrina* (Craw, 1891)	Coccoidae (Hemiptera)	Vayssières et al. 2001
		*Encarsia diaspidicola* (Silvestri, 1909)	*Pseudaulacaspis pentagona* (Hemiptera: Diaspididae)	Quilici et al. 2003
		*Encarsia formosa* Gahan, 1924	Aleyrodidae (Hemiptera)	Vayssières et al. 2001
		*Encarsia lounsburyi* (Berlese & Paoli, 1916)	Coccoidae (Hemiptera)	Quilici et al. 2003
		*Encarsia luteola* Howard, 1895	*Bemisia* (Hemiptera: Aleyrodidae)	Vayssières et al. 2001
		*Encarsia nigricephala* Dozier, 1937	*Trialeurodes* (Hemiptera: Aleyrodidae)	Vayssières et al. 2001
		*Encarsia perniciosi* (Tower, 1913)	*Quadraspidiotus perniciosus*	Quilici et al. 2003
		*Encarsia sophia* (Girault & Dodd, 1915)	*Bemisia tabaci*, *Trialeurodes vaporarium* (Hemiptera: Aleyrodidae)	Vayssières et al. 2001
**APHELINIDAE**	**Coccophaginae**	*Encarsia tabacivora* Viggiani, 1985	Aleyrodidae (Hemiptera)	Vayssières et al. 2001
	**Eretmocerinae**	*Eretmocerus hayati* (Zolnerowich & Rose, 1998)	*Bemisia tabaci*, *Trialeurodes vaporarium* (Hemiptera: Aleyrodidae)	Vayssières et al. 2001
		*Eretmocerus mundus* (Mercet, 1931)	*Bemisia argentifolii*, *Bemisia tabaci*,*Trialeurodes vaporarium* (Hemiptera: Aleyrodidae)	Vayssières et al. 2001
**CHALCIDIDAE**	**Chalcidinae**	*Brachymeria nephantidis* Gahan, 1930	*Nephantis serinopa* (Lepidoptera: Autostichidae), Corcyra cephalonica (Lepidoptera: Pyralidae)	Ramage et al. 2011
		*Brachymeria podagrica* (Fabricius, 1787)	Lepidoptera and Diptera	Ramage et al. 2011
		*Dirhinus galesusaeformis* (Risbec, 1957)	Unknown	Risbec 1957
		*Dirhinus giffardii* (Silvestri, 1913)	Tephritidae (Diptera)	Vayssières et al. 2001
		*Dirhinus himalayanus* Westwood, 1836	Some Diptera in: Tephritidae, Muscidae and Sarcophagidae	Ramage et al. 2011
	**Epitraninae**	*Epitranus erythrogaster* Cameron, 1888	*Corcyra cephalonica* (Lepidoptera: Pyralidae)	Ramage et al. 2011
		*Epitranus evanioides* (Westwood, 1835)	Unknown	Ramage et al. 2011
	**Haltichellinae**	*Anthrocephalus crassipes* (Masi, 1940)	Unknown	Ramage et al. 2011
		*Anthrocephalus dividens* (Walker, 1860)	Unknown	Ramage et al. 2011
		*Hockeria fluvipes* Masi, 1917	Unknown	Ramage et al. 2011
		*Proconura eublemmae* (Steffan, 1951)	*Eublemma gayneri* (Lepidoptera: Erebidae)	Ramage et al. 2011
**ENCYRTIDAE**	**Encyrtinae**	*Ageniaspis citricola* Logvinovskaya, 1983	*Phyllocnistis citrella* (Lepidoptera: Gracillaridae)	Quilici et al. 2003
		*Ageniaspis fuscicallis* (Dalman, 1920)	*Yponomeuta malinellus* (Lepidoptera: Yponomeutidae), Acrolepia assectella (lepidoptera: Acrolepidae)	Quilici et al. 2003
		*Aloencyrtus obscuratus* (Waterston, 1917)	Coccidae (Hemiptera)	Quilici et al. 2003
		*Arrhenophagus chionaspidis* (Aurivillius, 1888)	*Pseudaulacaspis pentagona* (Hemiptera: Diaspididae)	Quilici et al. 2003
		*Cheiloneurus cyanonotus* (Waterston, 1917)	*Phenacoccus manihoti* (Hemiptera: Pseudococcidae)	Risbec 1957
		*Comperiella bifasciata* (Howard, 1906)	Diaspididae (Hemiptera)	Quilici et al. 2003
		*Copidosoma koelheri* (Blanchard, 1940)	*Phtorimea operculella*	Vayssières et al. 2001
		*Diaphorencyrtus aligarhensis* (Shafee, Alam & Agarwal, 1975)	*Diaphorina citri* (Hemiptera: Psyllidae)	Quilici et al. 2003
**ENCYRTIDAE**	**Encyrtinae**	*Habrolepsis rouxi* (Compere, 1936)	*Aonidiella aurantii* (Hemiptera, Diaspididae)	Quilici et al. 2003
		*Homalotylus eytelweinii* (Ratzeburg, 1844)	Coccinellidae (Coleoptera)	Delpoux et al. 2013
		*Metaphycus galbus* (Annecke, 1964)	*Protopulvinaria pyriformis* (Hemiptera, Coccidae)	Quilici et al. 2003
		*Microterys nietneri* (Motschulsky, 1859)	Coccidae (Hemiptera)	Quilici et al. 2003
		*Psyllaephagus pulvinatus* (Waterson, 1922)	Psyllidae (Hemiptera)	Aubert 1975
		*Syrphophagus africanus* (Gahan, 1932)	Hyperparasitoid of aphid parasitoid	Vayssières et al. 2001
	**Tetracneminae**	*Coccidoxenoides perminutus* (Girault, 1915)	*Planococcus ficus* (Hemiptera: Pseudococcidae)	Quilici et al. 2003
**EULOPHIDAE**	**Entedoninae**	*Chrysocharis bedius* (Walker, 1842)	*Liriomyza* spp. (Diptera: Agromyzidae)	Vayssières et al. 2001
		*Chrysocharis* sp.	*Liriomyza* spp. (Diptera: Agromyzidae)	Vayssières et al. 2001
		*Chrysonotomyia pulcherrima* (Kerrich, 1970)	*Procontarinia matteiana* (Diptera: Cecidomyiidae)	Quilici et al. 2003
		*Neochrysocharis formosus* (Westwood, 1833)	*Liriomyza* spp. (Diptera: Agromyzidae)	Vayssières et al. 2001
		*Neochrysocharis* sp.	*Liriomyza* spp. (Diptera: Agromyzidae)	Vayssières et al. 2001
		*Platocharis coffeae* (Ferrière, 1936)	*Leucoptera* spp. (Lepidoptera: Lyonetiidae)	Ferrière 1936
	**Eulophinae**	*Cirrospilus cinctiventris* (Ferrière, 1936)	*Leucoptera* spp. (*Lepidoptera*: *Lyonetiidae*)	Quilici et al. 2003
		*Cirrospilus crowei* (Kerrich, 1969)	*Phyllocnistis citrella* (Lepidoptera: Gracillaridae)	Quilici et al. 2003
		*Elachertus insularis* (Risbec, 1957)	Unknown	Risbec 1957
		*Elasmus masii* Ferrière, 1929	(Hyper)Parasitoid of Lepidoptera	Risbec 1957
		*Euplectrus bebourensis* Risbec, 1957	Lepidoptera	Risbec 1957
		*Hamonia reunionensis* (Risbec, 1957)	Unknown	Risbec 1957
		*Hamonia sexdentata* (Risbec, 1957)	Unknown	Risbec 1957
		*Hamonia sylvatica* (Risbec, 1957)	Unknown	Risbec 1957
		*Hemiptarsenus varicornis* Girault, 1913	*Liriomyza* spp. (Diptera: Agromyzidae)	Vayssières et al. 2001
		*Notanisomorphella borborica* (Giard, 1903)	*Leucoptera* spp. (Lepidoptera: Lyonetiidae)	Quilici et al. 2003
		*Stenomesius belouvi* (Risbec, 1957)	Unknown	Risbec 1957
		*Stenomesius masii* (Risbec, 1957)	Unknown	Risbec 1957
	**Tetrastichinae**	*Aprostocetus ceroplastae* (Girault, 1916)	*Ceroplastes destructor* (Hemiptera, Coccidae)	Quilici et al. 2003
		*Aprostocetus toddaliae* (Risbec, 1958)	*Ceroplastes rusci* (Hemiptera, Coccidae)	Quilici et al. 2003
		*Gyrolasia hellburgi* (Risbec, 1957)	Unknown	Risbec 1957
		*Nesolynx phaeosoma* (Waterston, 1915)	*Epischnia beharella* (Lepidoptera: Pyralidae)	Quilici et al. 2003
**EULOPHIDAE**	**Tetrastichinae**	*Oomyzus sokolowskii* (Kurdjumov, 1912)	*Plutella xylostella* (Lepidoptera: Plutellidae)	Vayssières et al. 2001
		*Quadrastichus erythrinae* (Kim, 2004)	*Erythrina* (Fabales: Fabaceae)	Kim et al. 2004
		*Tamaraxia dryi* (Waterston, 1922)	Psyllidae (Hemiptera)	Etienne and Aubert 1980
		*Tamaraxia radiata* (Waterston, 1922)	Psyllidae (Hemiptera)	Aubert et al. 1979
		*Tetrastichus giffardianus* (Silvestri, 1915)	Tephritidae (Diptera)	Vayssières et al. 2001
		*Tetrastichus gyrolasiaeformis* (Risbec, 1957)	Unknown	Risbec 1957
		*Tetrastichus sesamiae* (Risbec, 1951)	Lepidoptera	Risbec 1957
**EUPELMIDAE**	**Eupelminae**	*Eupelmus* sp.	Extremely polyphagous	Vayssières et al. 2001
**EURYTOMIDAE**	**Eurytominae**	*Eurytoma ivohibei* (Risbec, 1957)	Unknown	Risbec 1957
		*Eurytoma reunionensis* (Risbec, 1957)	Unknown	Risbec 1957
**ORMYRIDAE**		*Ormyrus australis* Westwood, 1832	Unknown	Risbec 1957
**PTEROMALIDAE**	**Eunotinae**	*Mesopeltita truncatipennis* (Waterston, 1917)	Coccidae (Hemiptera)	Quilici et al. 2003
		*Moranila californica* (Howard, 1881)	*Saissetia oleae*, *Ceroplastes floridensis* (Hemiptera, Coccidae)	Quilici et al. 2003
	**Miscogasterinae**	*Halticoptera* sp.	*Melanagromyza phaseoli*, *Liriomyza* spp. (Diptera: Agromyzidae)	Vayssières et al. 2001
	**Pteromalinae**	*Muscidifurax raptor* (Girault & Sanders, 1910)	Muscidae (Diptera)	Quilici et al. 2003
		*Muscidifurax uniraptor* (Kogan &Legner, 1970)	Muscidae (Diptera)	Quilici et al. 2003
		*Pachyneuron* sp.	Extremely polyphagous	Vayssières et al. 2001
		*Pachyneuron longiradius* (Silvestri, 1915)	Coccinellidae (Coleoptera)	Delvare unpublished
		*Ploskana tenuis* (Boucek, 1976)	Tephritidae (Diptera)	Vayssières et al. 2001
**PTEROMALIDAE**	**Pteromalinae**	*Pteromalus* sp.	Extremely polyphagous	Vayssières et al. 2001
		*Spodophagus lepidopterae* (Risbec, 1952)	Noctuidae (Lepidoptera)	van Noort 2013
		*Trichomalopsis* sp.	Muscidae (Diptera)	Vayssières et al. 2001
	**Spalangiinae**	*Spalangia endius* (Walker, 1839)	*Musca domestica* (Diptera: Muscidae), *Dacus ciliatus* (Diptera: Tephritidae)	Vayssières et al. 2001
		*Spalangia gemina* Boucek, 1963	Muscidae, Tephritidae (Diptera)	Vayssières et al. 2001
		*Spalangia seyrigi* (Risbec, 1952)	*Dacus ciliatus* (Diptera: Tephritidae)	Vayssières et al. 2001
	**Sycoecinae**	*Seres bouceki* (Wiebes, 1981)	*Ficus reflexa reflexa* (Moraceae)	Wiebes 1981
	**Sycoryctinae**	*Apocrypta perplexa* Coquerel, 1855	*Ficus mauritiana* (Moraceae)	Coquerel 1855
		*Sycoryctes anceps* Wiebes, 1981	*Ficus densifolia* (Moraceae)	Wiebes 1981
		*Sycoryctes caelebs* Wiebes, 1975	*Ficus densifolia* (Moraceae)	Wiebes 1975
		*Sycoryctes comparabilis* Wiebes, 1981	*Ficus densifolia* (Moraceae)	Wiebes 1981
		*Sycoryctes remus* Wiebes, 1981	*Ficus reflexa reflexa* (Moraceae)	Bouček et al. 1981
		*Sycoscapter gibbus* Saunders, 1883	*Ficus politoria* and *Ficus lateriflora* (Moraceae)	Saunders 1883
		*Sycoscpater tibialis* Wiebes, 1981	*Ficus rubra* (Moraceae)	Wiebes 1981
		*Philotrypesis cnephaea* Wiebes, 1981	*Ficus reflexa reflexa* (Moraceae)	Wiebes 1981
		*Watshamiella fictitia* Wiebes, 1981	*Ficus rubra* (Moraceae)	Wiebes 1981
		*Watshamiella lucens* Wiebes, 1981	*Ficus densifolia* (Moraceae)	Wiebes 1981
**SIGNIPHORIDAE**	–	*Chartocerus* sp.	Coccinellidae (Coleoptera)	Delvare unpublished
**TORYMIDAE**	**Megastigminae**	*Megastigmus transvaalensis* (Hussey, 1956)	Anacardiaceae (Sapindales)	Habeck et al. 1989
**TRICHOGRAMMATIDAE**	**Trichogrammatinae**	*Trichogramma chilonis* (Ishii, 1941)	*Plutella xylostella* (Lepidoptera: Plutellidae)	Vayssières et al. 2001

**Table 3.** List of the Cynipoidea, Chrysidoidea, Platygastroidea, Diaprioidea, Ceraphronoidea and Evanioidea recorded from Reunion Island.

Superfamilly	Familly	Subfamilly	Genus / Species	General host information	Recorded from
**CYNIPOIDEA**	**FIGITIDAE**	**Eucoilinae**	*Aganaspis daci* (Weld, 1951)	Tephritidae (Diptera)	Etienne 1975
			*Leptopilina freyae* Allemand & Nordlander, 2003	*Drosophila* ssp.	Allemand et al. 2003
			*Leptopilina guineanensis* Allemand & Nordlander, 2003	*Drosophila* ssp.	Allemand et al. 2003
			*Leptopilina orientalis* Allemand & Nordlander, 2003	*Drosophila* ssp.	Allemand et al. 2003
			*Leptopilina victoriae* Nordlander, 1980	*Drosophila* ssp.	Allemand et al. 2003
			*Rhoptromeris bupalus* Quinlan, 1986	Chloropidae (Diptera)	Quinlan 1986
			*Rhoptromeris crito* Quinlan, 1986	Chloropidae (Diptera)	Quinlan 1986; Madl and Achterberg 2014
**CHRYSIDOIDEA**	**BETHYLIDAE**	**Bethylinae**	*Goniozus indicus* (Ashmead, 1903)	Pyralidae, Olethreutidae (Lepidoptera)	Polaszek et al. 1994
	**CHRYSIDIDAE**	**Amiseginae**	*Senesega attiei* (Kimsey, 2005)	Phasmatodea	Kimsey 2005
		**Chrysidinae**	*Chrysis gheudei* [1] Guérin-Méneville, 1842	Vespidae and Sphecidae (Hymenoptera)	Azevedo et al. 2010
**CHRYSIDOIDEA**	**CHRYSIDIDAE**	**Chrysidinae**	*Praestochrysis lusca* (Fabricius, 1804)	*Chalybion madecassumSceliphron fuscum* (Hymenoptera: Apoidea: Sphecidae)	Bordage 1912
	**DRYINIDAE**	**Anteoninae**	*Anteon reunionense* (Olmi, 1987)	Cicadellidae (Hemiptera)	Olmi 1987
		**Gonatopodinae**	*Gonatopus similis* Brues, 1906	*Dicranotropis muiri*, *Perkinsiella saccharicida* (Hemiptera: Delphacidae)	Olmi 1987a
**PLATYGASTROIDEA**	**PLATYGASTRIDAE**	**Platygastrinae**	*Leptacis risbeci* (Masner, 1960)	Cecidomyidae (Diptera)	van Noort 2013
			*Synopeas pauliani* (Risbec, 1957)	Cecidomyidae (Diptera)	Risbec 1957
	**SCELIONIDAE**	**Scelioninae**	*Macroteleia insularis* (Risbec, 1957)	Unknown	Risbec 1957
			*Styloteleia gibbosa* (Risbec, 1957)	Unknown	Risbec 1957
		**Telenominae**	*Telenomus busseolae* Gahan, 1922	Pyralidae (Lepidoptera)	Rao and Nagaraja 1969
**DIAPRIOIDEA**	**DIAPRIIDAE**	**Diapriinae**	*Trichopria belouvi* (Risbec, 1957)	Tachinidae, Tephritidae (Diptera)	Risbec 1957; Notton 2004; Madl 2015
			*Trichopria inconspicua* (Kieffer, 1905)	Tachinidae, Tephritidae (Diptera)	Huggert 1977; Madl 2015
			*Trichopria jeanneli* Notton, 2004	Tachinidae, Tephritidae (Diptera)	Risbec 1957; Madl 2015
			*Trichopria scotti* (Kieffer, 1912)	Tachinidae, Tephritidae (Diptera)	Risbec 1957
			*Trichopria variabilis* (Risbec, 1950)	Tachinidae, Tephritidae (Diptera)	Risbec 1957
**CERAPHRONOIDEA**	**CERAPHONIDAE**	-	*Aphanogmus fijensis* (Ferrière, 1933)	*Cotesia sesamiae* (Braconidae)	Polaszek and LaSalle 1995; Madl 2015
			*Ceraphron amphimelas* (Dessart 1989)	Hemiptera: Diaspididae	Dessart 1984; Madl 2015
	**MEGASPILIDAE**		*Dendrocerus wollastoni* (Dodd, 1920)	Coccinellidae (Coleoptera)	Delvare unpublished
			*Dendrocerus* sp.	Coccinellidae (Coleoptera)	Delvare unpublished
**EVANIOIDEA**	**EVANIIDAE**	-	*Evania appengaster* (Linnaeus, 1758)	Blattidae (Blattodea)	Madl and Ganeshan 2008
			*Prosevania erythrosoma* [2] (Schletterer, 1886)	*Blatta orientalis* (Blattodea: Blattidae)	Madl and Ganeshan 2008

[1] Probably a misidentification of *Chrysis lincea* (Azevedo et al. 2010) [2] Recorded from Rodrigues and Mauritious, not (yet) from Reunion Island
